# Targeted reduction of cholesterol uptake in cholesterol-addicted lymphoma cells blocks turnover of oxidized lipids to cause ferroptosis

**DOI:** 10.1074/jbc.RA120.014888

**Published:** 2020-11-24

**Authors:** Jonathan S. Rink, Adam Yuh Lin, Kaylin M. McMahon, Andrea E. Calvert, Shuo Yang, Tim Taxter, Jonathan Moreira, Amy Chadburn, Amir Behdad, Reem Karmali, C. Shad Thaxton, Leo I. Gordon

**Affiliations:** 1 Division of Hematology and Oncology, Department of Medicine, Northwestern University Feinberg School of Medicine, Chicago, Illinois, USA; 2 Simpson Querrey Institute for BioNanotechnology, Northwestern University, Chicago, Illinois, USA; 3 Robert H Lurie Comprehensive Cancer Center of Northwestern University, Chicago, Illinois, USA; 4 Department of Urology, Northwestern University Feinberg School of Medicine, Chicago, Illinois USA; 5 Department of Pathology, Northwestern University Feinberg School of Medicine, Chicago, Illinois, USA; 6 Department of Pathology, Weill Cornell Medical Center, New York, New York, USA; 7 International Institute for Nanotechnology, Northwestern University, Evanston, Illinois, USA

**Keywords:** cholesterol, high-density lipoprotein (HDL), nanotechnology, lipid peroxidation, lymphoma, scavenger receptor type B1 (SCARB1), glutathione peroxidase 4 (GPX4), ferroptosis, ABC, activated B cell, ALCL, anaplastic large T cell lymphoma, ALK, anaplastic lymphoma kinase, AuNP, gold nanoparticles, BCR, B cell receptor, BL, Burkitt’s lymphoma, C11-BODIPY, 4,4-difluoro-5-(4-phenyl-1,3-butadienyl)-4-bora-3a,4a-diaza-s-indacene-3-undecanoic acid, DFO, deferoxamine, DHCR7, dehydrocholesterol reductase 7, DiI, 1,1’-dioctadecyl-3,3,3’,3’-tetramethylindocarbocyanine perchlorate, DLBCL, diffuse large B cell lymphoma, DPPC, 1,2-dipalmitoyl-sn-glycero-3-phosphocholine, FACS, fluorescent activated cell sorter, FBS, fetal bovine serum, FL, follicular lymphoma, HCM, hepatocyte culture media, Hepes, 4-(20hydroxyethyl)-1-piperasineethanesulfonic acid, GC, germinal center, GPX4, glutathione peroxidase 4, HDL, high-density lipoprotein, HDL NPs, HDL-like nanoparticles, HMGCS1, HMG-CoA synthase 1, HSD17B7, 17β-hydroxysteroid dehydrogenase type 7, IHC, immunohistochemistry, INSIG1, insulin induced gene-1, LDL, low-density lipoprotein, LDLR, LDL receptor, L-OH, lipid alcohols, L-OOH, lipid peroxides, MPER, mammalian protein extraction reagent, NF-kB, nuclear factor kappa-light-chain-enhancer of activated B cells, PBS, phosphate buffered saline, PDP PE, 1,2-dipalmitoyl-sn-glycero-3-phosphoethanolamine-N-[3-(2-pyridyldithio)propionate], PDX, patient-derived xenografts, PI, propridium iodide, SCARB1, scavenger receptor type B-1, SQLE, squalene epoxidase, SREBP-1a, sterol response element binding protein-1a, STR, short tandem repeat, TFF, tangential flow filtration

## Abstract

Normal human cells can either synthesize cholesterol or take it up from lipoproteins to meet their metabolic requirements. In some malignant cells, de novo cholesterol synthesis genes are transcriptionally silent or mutated, meaning that cholesterol uptake from lipoproteins is required for survival. Recent data suggest that lymphoma cells dependent upon lipoprotein-mediated cholesterol uptake are also subject to ferroptosis, an oxygen- and iron-dependent cell death mechanism triggered by accumulation of oxidized lipids in cell membranes unless the lipid hydroperoxidase, glutathione peroxidase 4 (GPX4), reduces these toxic lipid species. To study mechanisms linking cholesterol uptake with ferroptosis and determine the potential role of the high-density lipoprotein (HDL) receptor as a target for cholesterol depleting therapy, we treated lymphoma cell lines known to be sensitive to the reduction of cholesterol uptake with HDL-like nanoparticles (HDL NPs). HDL NPs are a cholesterol-poor ligand that binds to the receptor for cholesterol-rich HDLs, scavenger receptor type B1 (SCARB1). Our data reveal that HDL NP treatment activates a compensatory metabolic response in treated cells toward increased de novo cholesterol synthesis, which is accompanied by nearly complete reduction in expression of GPX4. As a result, oxidized membrane lipids accumulate, leading to cell death through a mechanism consistent with ferroptosis. We obtained similar results *in vivo* after systemic administration of HDL NPs in mouse lymphoma xenografts and in primary samples obtained from patients with lymphoma. In summary, targeting SCARB1 with HDL NPs in cholesterol uptake–addicted lymphoma cells abolishes GPX4, resulting in cancer cell death by a mechanism consistent with ferroptosis.

Despite long-term remission observed in some patients with lymphoma, greater than one-third of patients with the most common subtype, diffuse large B cell lymphoma (DLBCL), will relapse or have disease that is refractory to primary treatment (1, 2, 3). This is especially the case for patients in high-risk groups identified by molecular and clinical prognostic factors (4, 5). Experimental therapies for these patients, including immunotherapy and cell-based therapies, have modest success rates, high cost, and toxicity (6, 7). Thus, there is a significant need for a new therapeutic approach.

Recently, DLBCL was identified as a cancer type particularly sensitive to cell death by ferroptosis (8). Ferroptosis is an iron-dependent form of necroptosis characterized by accumulation of cell membrane lipid and cholesterol peroxides that results from the targeted inhibition of glutathione peroxidase 4 (GPX4) (9, 10, 11). Cells become vulnerable to ferroptosis after GPX4 inhibition because the enzyme reduces and detoxifies lipid peroxides (L-OOH) by converting them to corresponding lipid alcohols (L-OH) (8, 10). Malignant cells under oxidative stress are significantly more sensitive to ferroptosis because of higher levels of reactive oxygen species and a reliance on GPX4 activity to mitigate toxic L-OOH accumulation (12, 13, 14). This finding has driven significant interest in the development of treatments to induce ferroptosis in cancer cells. Small-molecule inhibitors of GPX4 have been developed and tested, but they are toxic and lack specificity (15), which limits *in vivo* use and clinical relevance.

Data suggest a link between cellular cholesterol uptake and ferroptosis. For instance, removal of lipoproteins from media used to culture ALK^+^ anaplastic large T cell lymphoma (ALK^+^ ALCL) cell lines (SR-786, SUDHL1) and a histiocytic lymphoma cell line (U937) induced ferroptosis (16). Analyses of these cell lines revealed that a loss of squalene epoxidase expression (*SQLE*, aka squalene monooxygenase, ALK^+^ ALCL cells), through hypermethylation of its promoter region, or 3-ketosteroid reductase expression (*HSD17B7*, U937 cells), through a mutational defect, rendered the cells auxotrophic for cholesterol. SQLE (17) and HSD17B7 (18) are enzymes required for de novo cellular cholesterol biosynthesis, which explains the cellular obligation for cholesterol uptake. In these cholesterol auxotrophic cells, studies have shown that inhibition of low-density lipoprotein (LDL)–mediated cholesterol uptake by reduction of expression of the LDL receptor (LDLR) reduced the viability of ALK^+^ ALCL cells and ALK^+^ ALCL patient-derived xenografts *in vivo* (16) and sensitized the cells to ferroptosis by GPX4 inhibitors (16). These data suggest that reduction of cellular cholesterol uptake renders cells sensitive to ferroptosis; however, the mechanism remains unclear.

Scavenger receptor type B-1 (SCARB1) is a high-affinity receptor for cholesterol-rich high-density lipoproteins (HDLs), which has been implicated as a target in human cancers based upon its role in cancer cell cholesterol uptake (19, 20, 21, 22, 23, 24, 25, 26, 27) and in membrane-anchored second messenger signaling pathways (23, 28, 29, 30, 31), among other factors (32, 33, 34, 35). Our group developed cholesterol-poor HDL-like nanoparticles (HDL NPs) to target and tightly bind SCARB1 to prevent the cellular uptake of cholesterol ester from HDLs (23, 26, 36). We have demonstrated that certain B cell lymphomas highly express SCARB1, and targeted treatment with the HDL NPs potently induces *in vitro* and *in vivo* cell death by inhibiting cholesterol ester uptake and reducing cell cholesterol (23, 26, 27). We have shown that HDL NP–mediated inhibition of cholesterol ester uptake in highly sensitive B cell lymphoma cells, including Burkitt’s lymphoma (BL) (*e.g.* Ramos cell line) and DLBCL (*e.g.* SUDHL4 cell line), is accompanied by compensatory increases in the expression of genes required for de novo cholesterol synthesis (23). We have shown that lymphoma cells not initially sensitive to HDL NP–mediated cholesterol depletion, including activated B cell (ABC) lymphoma (*e.g.* TMD8 and HBL-1 cell lines), exhibit a high baseline expression of de novo cholesterol biosynthesis genes and higher cholesterol content relative to the highly sensitive lymphoma cell lines (23). These data are consistent with the report by Chen *et al* showing that more active second messenger pathways downstream of the B cell receptor (BCR) result in de novo cholesterol biosynthesis (37). In the case of the ABC lymphoma cells, our data show that cell death can be induced with a synergistic combination of HDL NPs and targeted inhibitors of downstream BCR tyrosine kinases (23). Thus, targeting of SCARB1 by HDL NPs with reduction of cell cholesterol uptake and compensatory increase in de novo cholesterol biosynthesis is a potential translational model for treatment of cholesterol uptake–dependent lymphoma.

Accordingly, our group explored whether HDL NP therapy targeting SCARB1 induced lymphoma cell death through a mechanism involving GPX4 and ferroptosis. Initially, we employed a gene microarray as an unbiased approach to study changes in gene expression caused by HDL NPs in a cholesterol uptake–dependent lymphoma cell line. These data revealed that HDL NPs obligate cellular expression of de novo cholesterol biosynthesis genes, which is accompanied by reduced GPX4 expression. Our data show that reduced GPX4 expression leads to an increase in membrane-oxidized lipids and cell death through a mechanism consistent with ferroptosis in cell lines, in an *in vivo* xenograft model, and in primary samples obtained from patients with lymphoma.

## Results

### HDL NPs bind to SCARB1 in lymphoma and induce cell death

We previously demonstrated that Ramos and SUDHL4 cells, well-studied models of BL and germinal center DLBCL (GC DLBCL), respectively, highly express SCARB1 (26). Also, our prior data showed that HDL NPs target SCARB1 in SUDHL4 and Ramos cells, which resulted in cellular cholesterol depletion and profound *in vitro* and *in vivo* cell death (23, 26). Here, we verified the requirement of SCARB1 as a target of HDL NPs in these lymphoma cells using an anti-SCARB1 blocking antibody (Ab) and fluorescently labeled HDL NPs. HDL NPs were fluorescently labeled using the intercalating dye 1,1’-dioctadecyl-3,3,3’,3’-tetramethylindocarbocyanine perchlorate (DiI), as previously described (23, 38). Ramos and SUDHL4 cells were treated with DiI HDL NPs for 2 h followed by flow cytometric analysis. DiI HDL NP treatment increased the fluorescent signal in both cell lines (Fig. S1, *A* and *B*), which was reduced by cotreatment with the SCARB1 blocking Ab (1:100 dilution), but not by an isotype control Ab (Fig. S1, *A* and *B*). DiI HDL NP treatment of Jurkat cells, a SCARB1-negative T cell leukemia/lymphoma cell line, did not result in increased fluorescent signal over baseline (Fig. S1, *C*), confirming that the HDL NPs target SCARB1 in B cell lymphoma. We next investigated whether other BL and GC DLBCL cell lines expressed SCARB1 and if they were sensitive to HDL NP–induced cell death. The BL cell lines Raji, Daudi, and Namalwa and the GC DLBCL cell line SUDHL6 all expressed SCARB1 (Fig. S2, *A*). HDL NP treatment potently induced cell death in all SCARB1-positive B cell lymphoma cell lines, while having no effect on the SCARB1-negative Jurkat cell line (Fig. S2, *B*). Inhibition of HDL NP binding to SCARB1 using an SCARB1 blocking Ab protected all SCARB1-positive cell lines from HDL NP–induced cell death in a dose-dependent manner, and the SCARB1 blocking Ab did not result in cell death when added in isolation (Fig. S2, *C–H*). The isotype control Ab had no effect on HDL NP–induced cell death (data not shown).

Because native cholesterol-rich HDLs have been reported to bind both SCARB1 and LDLR (39), we tested if HDL NPs also bound to the LDLR in the Ramos and SUDHL4 cells and if HDL NP–induced cell death was specific to their binding SCARB1. First, addition of an LDLR blocking Ab (40, 41) reduced binding of HDL NPs to Ramos and SUDHL4 cells, however, not to the extent observed when the SCARB1 blocking Ab was added (Fig. S3, *A* and *B*). Also, the combination of SCARB1 blocking Ab and LDLR blocking Ab resulted in a statistically significant reduction in DiI HDL NP binding compared with addition of the SCARB1 blocking Ab alone (Fig. S3, *A* and *B*). Second, from a functional standpoint, addition of the LDLR blocking Ab had no effect on HDL NP–induced cell death in Ramos or SUDHL4 cells (Fig. S3, *C* and *D*) and the combination of both blocking antibodies provided no additional protection against HDL NP–induced cell death compared with the SCARB1 blocking Ab group (Fig. S3, *C* and *D*).

### HDL NP binding to SCARB1 increases de novo cholesterol biosynthesis and reduces GPX4 expression in lymphoma

**Figure 1:**
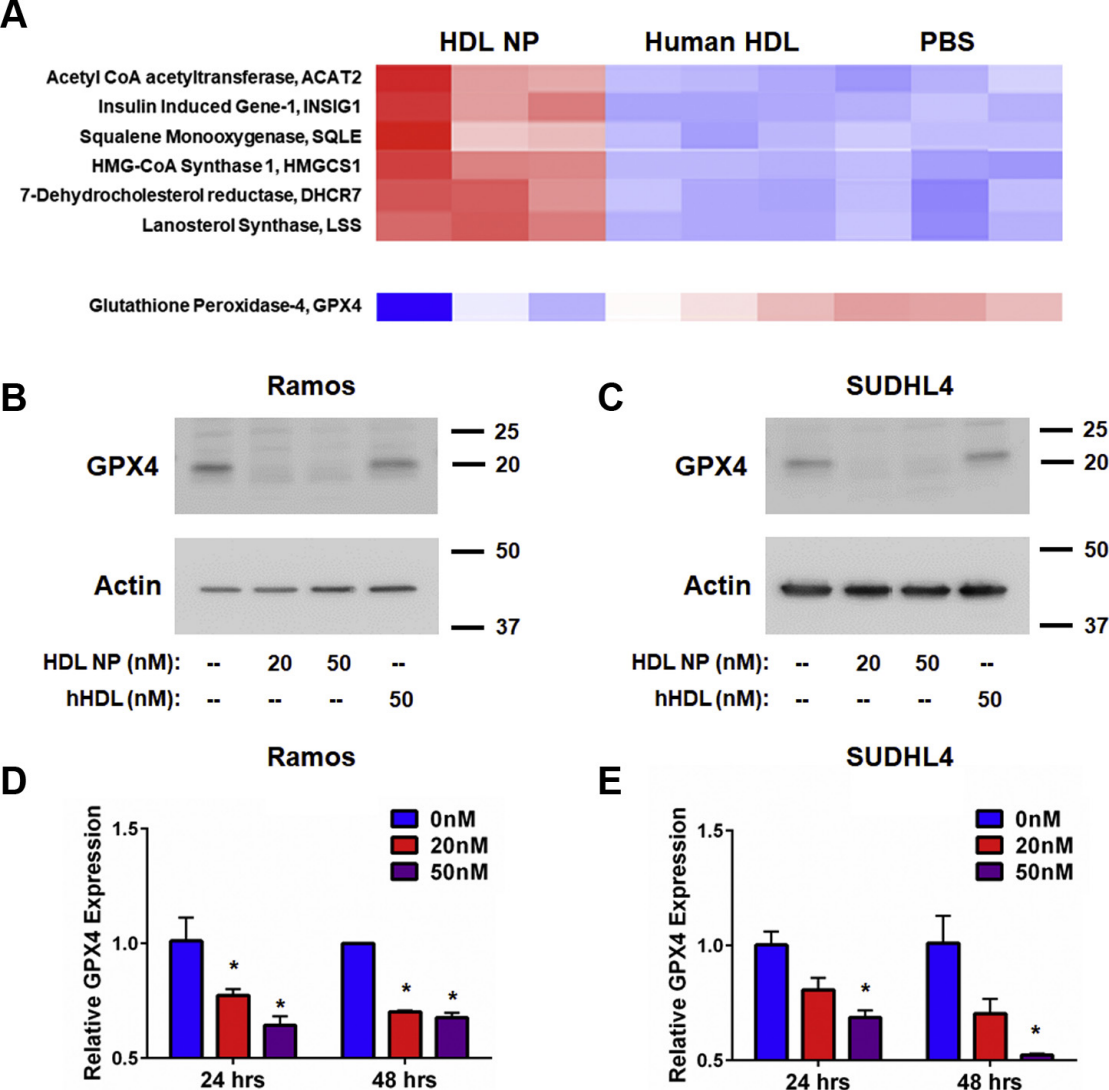
**HDL NPs increase expression of de novo cholesterol synthesis genes and reduce expression of GPX4.***A*. Select gene microarray results from Ramos cells treated with HDL NPs or human HDL (hHDL) for 48 h. *B* and *C*. Western blot analysis of GPX4 expression in Ramos (*B*) and SUDHL4 (*C*) cells treated with HDL NPs or hHDL for 48 h. β-Actin was used as a loading control. *D* and *E*. RT-qPCR analysis for GPX4 expression in Ramos (*D*) and SUDHL4 (*E*) cells treated with HDL NPs for 24 or 48 h. ∗*p* < 0.05 vs 0 nM. HDL NPs, high-density lipoprotein–like nanoparticles.

We performed an unbiased gene array study in Ramos cells to broadly screen for changes in gene expression that may explain increased cell death induced by HDL NPs. As a control, we treated cells with either phosphate buffered saline (PBS) or an equimolar concentration of cholesterol-rich human HDLs. Our data show that HDL NP treatment induces a robust increase in the expression of genes involved in de novo cholesterol biosynthesis, as anticipated (23) (Fig. 1, *A*; Fig. S4). Western blot data were obtained to confirm the increase in expression of the de novo cholesterol biosynthesis gene, HMGCS1, after treatment with HDL NPs (Fig. S5). With regard to genes whose expression was significantly reduced, importantly, we found that *GPX4* was suppressed upon HDL NP treatment (Fig. 1, *A*; Fig. S4). We confirmed decreased GPX4 expression using Western blot analysis and conventional RT-qPCR. Ramos and SUDHL4 cells were treated with HDL NPs for up to 48 h. We found that HDL NP treatment profoundly reduced expression of GPX4 in both cell lines relative to PBS control at both the protein (Fig. 1, *B* and *C*) and mRNA level (Fig. 1, *D* and *E*). By contrast, treatment with an equimolar concentration of cholesterol-rich HDLs did not alter GPX4 protein or gene expression (Fig. 1, *A–C*).

### HDL NP induces ferroptosis in B cell lymphoma cell lines by reducing GPX4

**Figure 2:**
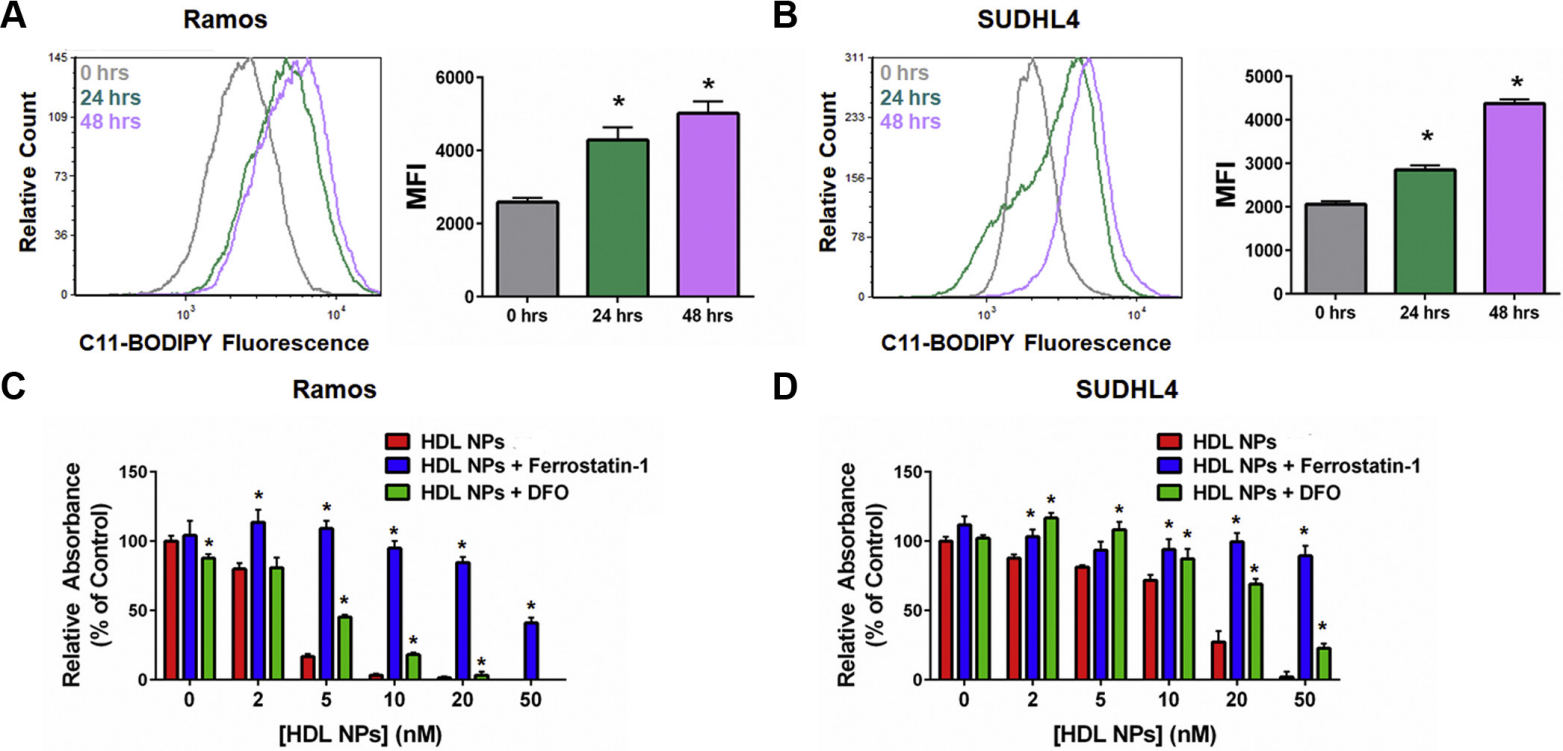
**HDL NPs induce ferroptosis in B cell lymphoma cells.***A* and *B*. Flow cytometric analysis of C11-BODIPY fluorescence in Ramos (*A*) and SUDHL4 (*B*) cells treated with HDL NPs (50 nM) over time. ∗*p* < 0.05 vs. 0 h. *C* and *D*. Cell viability (MTS) assays for Ramos (*C*) and SUDHL4 (*D*) cells treated with HDL NPs, ferrostatin-1 (1 μM), and/or DFO (1 μM) for 72 h. ∗*p* < 0.05 vs HDL NPs. DFO, deferoxamine; HDL NPs, high-density lipoprotein–like nanoparticles.

Stockwell *et al*. (10) proposed two metrics to distinguish ferroptosis from apoptosis and other forms of cell death: (1) Cell death correlates with an increase in oxidized membrane lipids quantified by using C11-BODIPY, a lipophilic fluorescent dye that has a unique spectral signature when oxidized and is used to measure lipid peroxidation, and flow cytometry; and (2) Cell death can be reduced by addition of a lipophilic antioxidant (*e.g.,* ferrostatin-1) or an iron chelator (*e.g.,* deferoxamine [DFO]). Using these parameters, we investigated whether HDL NPs induced ferroptosis in Ramos and SUDHL4 cells. In both cell lines, HDL NP treatment led to a dose-dependent increase in C11-BODIPY signal over time (Fig. 2, *A* and *B*). Next, Ramos and SUDHL4 cells were cultured with HDL NPs in the presence of either ferrostatin-1 or DFO and assayed for cell viability. Addition of ferrostatin-1 and DFO significantly inhibited HDL NP–induced cell death in Ramos (Fig. 2, *C*) and SUDHL4 (Fig. 2, *D*) cells.

To confirm the role of GPX4 in mediating HDL NP–induced cell death, GPX4 was overexpressed in Ramos cells by transfecting an untagged GPX4 clone in a pCMV6-AC vector (Origene) via lentiviral infection. Following antibiotic selection, GPX4 overexpression was confirmed by Western blot and RT-qPCR (Fig. S6, *A* and *B*). As anticipated, overexpression of GPX4 led to higher GPX4 levels following HDL NP treatment compared with controls (Fig. S6, *C*) and protected B cell lymphoma cells from HDL NP–induced cell death compared with controls (Fig. S6, *D*).

Finally, to demonstrate the specificity of the ferroptosis phenotype to malignant as opposed to normal SCARB1-positive cells, we investigated whether HDL NP treatment led to changes in GPX4 expression or lipid peroxide accumulation in primary human hepatocytes. Previously, we demonstrated that primary human hepatocytes express SCARB1 but do not undergo cell death when treated with HDL NPs (26). Treatment of primary human hepatocytes for 72 or 120 h did not alter the expression of GPX4, as assayed by Western blot analysis (Fig. S7, *A*). Correspondingly, no change in lipid peroxide accumulation was observed with HDL NP treatment (Fig. S7, *B*).

### HDL NP targets SCARB1 and induces ferroptosis in cholesterol auxotrophic lymphoma cell lines

**Figure 3:**
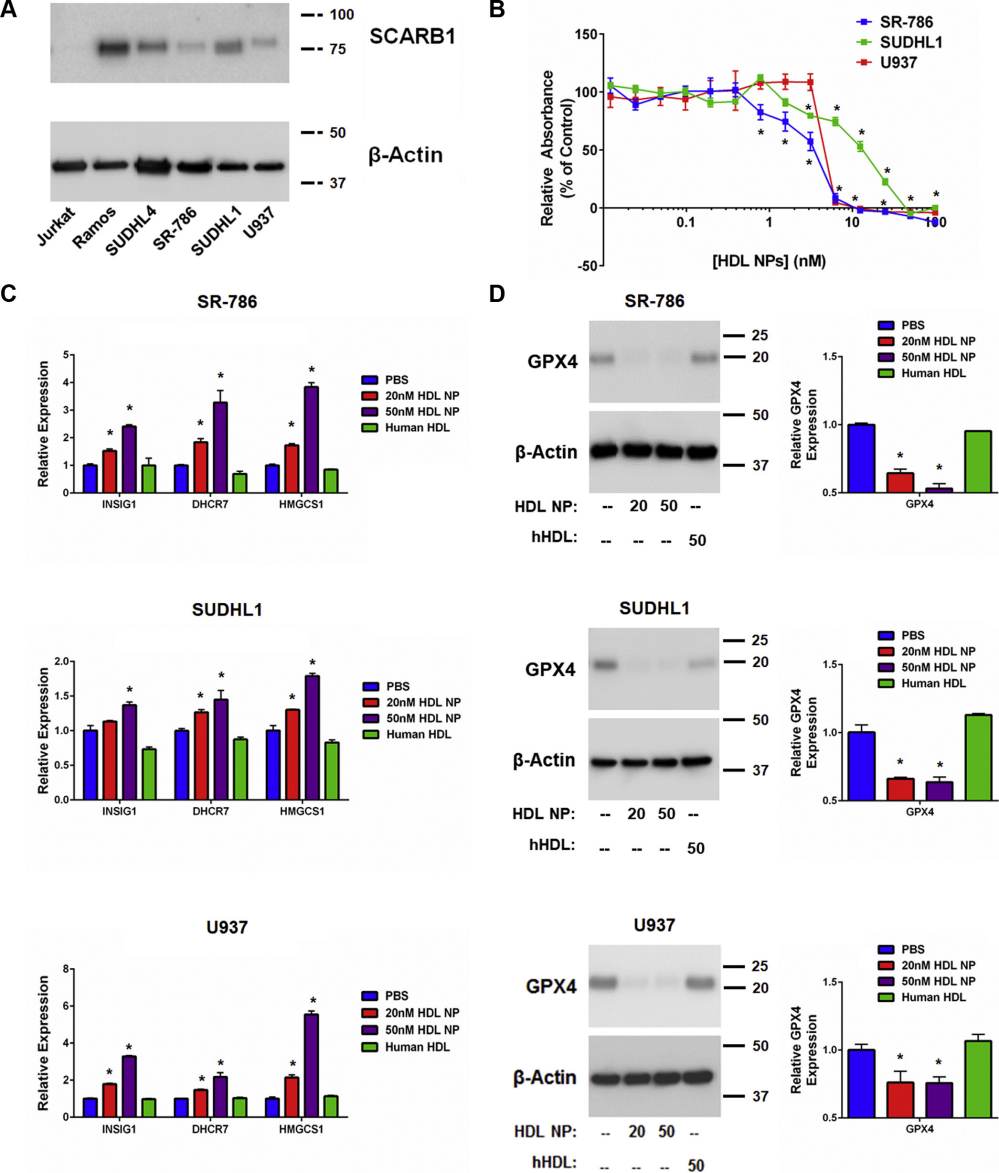
**Cholesterol auxotrophic cell lines express SCARB1, and HDL NP treatment reduces cell viability and alters gene expression.***A*. Western blot analysis of SCARB1 expression in SUDHL1, SR-786, and U937 cells. Ramos and Jurkat represent positive and negative controls for SCARB1 expression, respectively. β-Actin was used as a loading control. *B*. Cell death (MTS) assay of SR-786, SUDHL1, and U937 cells treated with HDL NPs for 120 h. ∗*p* < 0.05 vs PBS control. *C*. RT-qPCR analysis of cholesterol biosynthesis genes *INSIG1*, *DHCR7*, and *HMGCS1* in SR-786, SUDHL1, and U937 cells treated with HDL NPs (20 nM, 50 nM) or human HDL (50 nM) for 72 h. ∗*p* < 0.05 vs PBS control. *D*. Western blot (left) and RT-qPCR (right) analysis of GPX4 expression in SR-786, SUDHL1, and U937 cells following 72-h treatment with HDL NPs (20 nM, 50 nM) or human HDL (50 nM). ∗*p* < 0.05 vs PBS control. HDL NPs, high-density lipoprotein–like nanoparticles; PBS, phosphate buffered saline.

Recently, a number of cell lines were found to be auxotrophic for cholesterol, including the cell lines SR-786 (ALK^+^ ALCL), SUDHL1 (ALK^+^ ALCL), and U937 (isolated from histiocytic lymphoma, but of myeloid lineage), among others (16). The ALK^+^ ALCL cells were identified based upon reduced viability when cultured in lipoprotein-deficient serum, and the cell death phenotype was rescued by addition of cholesterol-rich LDL or free cholesterol. However, because cells can uptake cholesterol by cholesterol-rich HDL binding to SCARB1, we studied expression of SCARB1 in ALK^+^ ALCL and U937 cells. We found SCARB1 expression in SR-786, SUDHL1, and U937 cells (Fig. 3, *A*) accompanied by cell death after treatment with HDL NPs (Fig. 3, *B*).

Similar to B cell lymphoma cells, addition of the SCARB1 blocking Ab reduced binding of fluorescent DiI HDL NPs to SR-786, SUDHL1, and U937 cells and protected against HDL NP–induced cell death (Fig. S8, *A–C*). Addition of the LDLR blocking Ab also reduced DiI HDL NP binding, however, not to the extent seen with addition of the SCARB1 blocking Ab, and it had no impact on HDL NP–induced cell death (Fig. S8, *D–F*).

Analyses of the following select de novo cholesterol biosynthesis genes in SR-786, SUDHL1, and U937 cells after HDL NP treatment demonstrated increased expression: INSIG1 (insulin-induced gene-1), DHCR7 (dehydrocholesterol reductase 7) and HMGCS1 (HMG-CoA synthase 1) (Fig. 3, *C*). Similar to the B cell lymphoma cell lines, HDL NP treatment potently reduced GPX4 expression at both the RNA and protein levels (Fig. 3, *D*).

**Figure 4:**
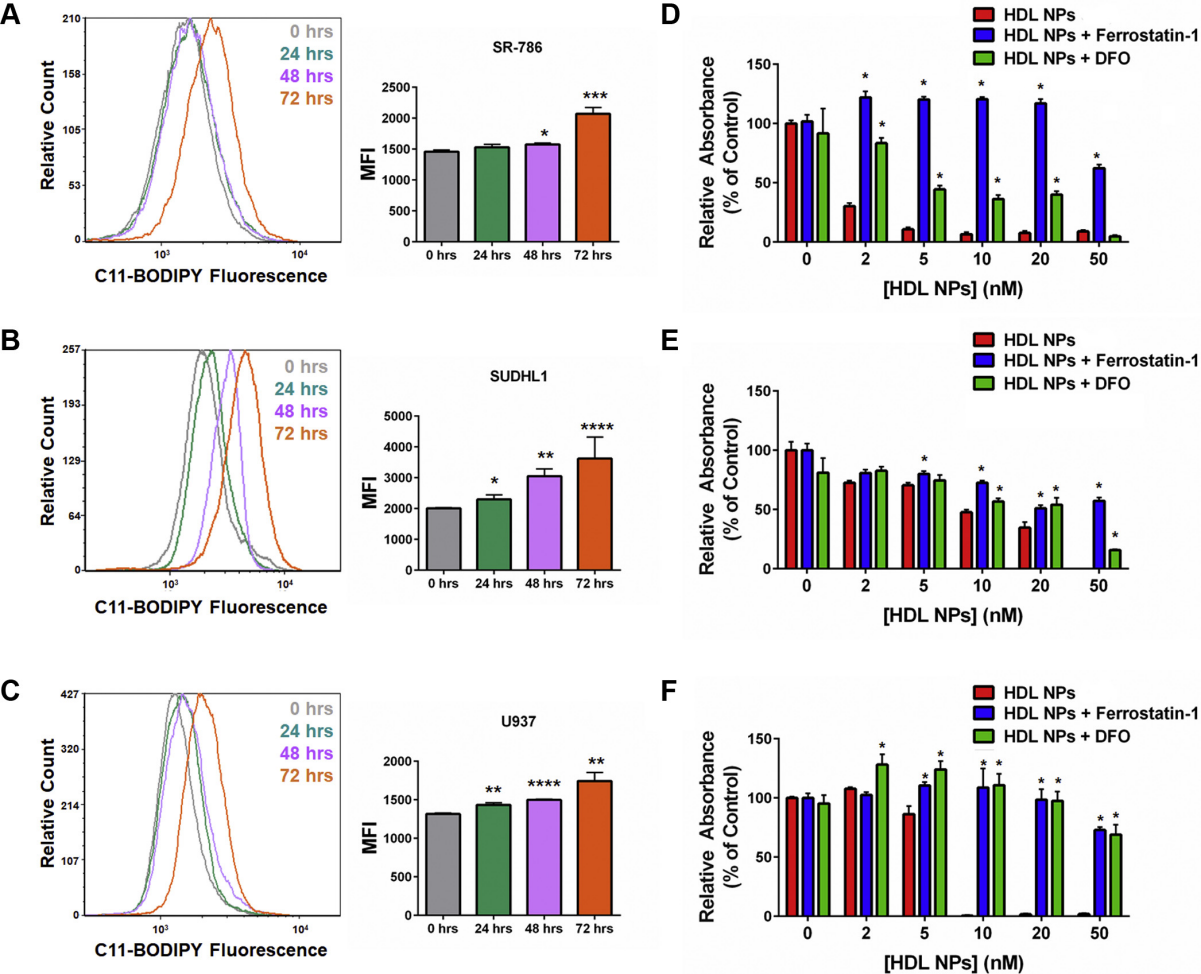
**HDL NPs induce ferroptosis in cholesterol auxotrophic cell lines.***A–C*. Flow cytometric analysis for C11-BODIPY fluorescence in SR-786 (*A*), SUDHL1 (*B*), and U937 (*C*) cells treated with HDL NPs (50 nM) over 72 h. ∗*p* < 0.05 vs 0 h. ∗∗*p* < 0.01 vs. 0 h. ∗∗∗*p* < 0.005 vs. 0 h. ∗∗∗∗*p* < 0.001 vs. 0 h. *D–F*. Cell death (MTS) assays for SR-786 (*D*), SUDHL1 (*E*), and U937 (*F*) cells treated with HDL NPs, ferrostatin-1 (1 μM), and/or DFO (1 μM) for 120 h. ∗*p* < 0.05 vs HDL NPs. DFO, deferoxamine; HDL NPs, high-density lipoprotein–like nanoparticles.

Corresponding to the decrease in GPX4 expression, HDL NPs induced accumulation of lipid peroxides in SR-786, SUDHL1, and U937 cells, as measured by C11-BODIPY flow cytometry (Fig. 4, *A–C*). Addition of the ferroptosis inhibitors ferrostatin-1 and DFO rescued SR-786, SUDHL1, and U937 cells from HDL NPinduced cell death (Fig. 4, *D–F*), confirming cell death by ferroptosis. Taken together, these data indicate that inhibition of cholesterol ester uptake by HDL NP binding to SCARB1 induces ferroptosis in previously identified cholesterol auxotrophic cell lines.

### HDL NP induces ferroptosis *in vivo*

**Figure 5:**
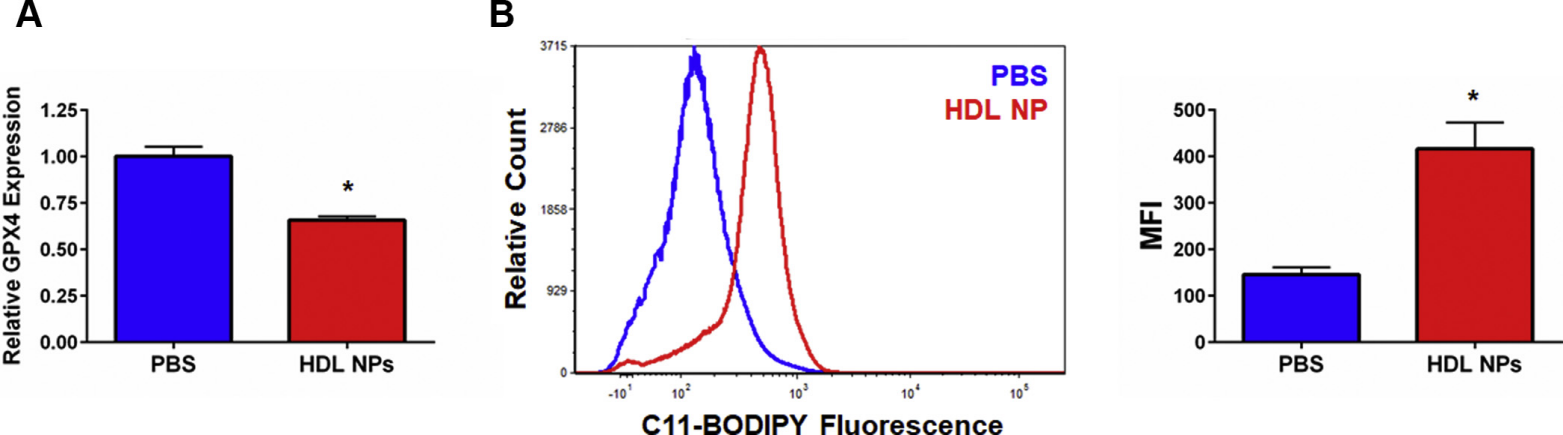
**HDL NPs induce ferroptosis *in vivo*.***A*. RT-qPCR analysis for GPX4 from SUDHL4 tumor xenografts isolated following HDL NP treatment. ∗*p* < 0.05 vs PBS control. *B*. Flow cytometric analysis of C11-BODIPY fluorescence in SUDHL4 tumor xenograft cells following HDL NP treatment. Flow histogram displaying C11-BODIPY fluorescence (left). Median fluorescent intensity (MFI) graph (right). ∗*p* < 0.05 vs PBS control. HDL NPs, high-density lipoprotein–like nanoparticles; PBS, phosphate buffered saline.

We previously reported that HDL NPs specifically target and significantly reduce tumor burden in xenograft models using SUDHL4 and Ramos cells (23, 26). To determine if systemic HDL NP treatment reduces GPX4 expression and increases lipid peroxide accumulation in tumor cells *in vivo*, we established SUDHL4 tumor xenografts (∼100 mm^3^ in volume) in severe combined immunodeficiency–beige mice. The mice were then treated with PBS or HDL NPs (100 μl of 1 μM HDL NP, 3X/week for 1 week, i.v.). Following treatment, tumors were resected and GPX4 expression and lipid peroxide accumulation were quantified by RT-qPCR and C11-BODIPY staining, respectively. HDL NP treatment led to a downregulation of GPX4 as measured by RT-qPCR compared with PBS controls (Fig. 5, *A*), which correlated with an increase in membrane lipid peroxide accumulation (Fig. 5, *B*). No adverse side effects were observed after systemic administration of HDL NPs. These data show that HDL NPs induce molecular changes consistent with ferroptosis in the SUDHL4 flank tumor xenograft model of lymphoma previously demonstrated to be sensitive to HDL NP therapy (23).

### *In vivo* SCARB1 expression from archival patient specimens

Our *in vitro* data suggest that SCARB1 is a useful biomarker to identify patients with lymphoma where this targeted approach might be successful. We have previously reported, using microarray data from a cohort of patients with DLBCL from a single institution, that SCARB1 expression was increased in DLBCL compared with normal naïve and memory B cells (26). In order to confirm SCARB1 expression from archival primary tissue, we performed immunohistochemistry (IHC) staining of formalin-fixed paraffin-embedded DLBCL patient samples (Fig. S9, *A*). In addition, SCARB1 expression in follicular lymphoma (FL) was also investigated (Fig. S9, *A*). Normal liver and thymus tissue were used as positive and negative controls, respectively. SCARB1 expression was observed in representative samples of both DLBCL and FL samples, providing evidence that this is a viable target in patients (Fig. S9, *A*).

### HDL NPs induce cell death in primary lymphoma cells obtained from patients with lymphoma

**Figure 6:**
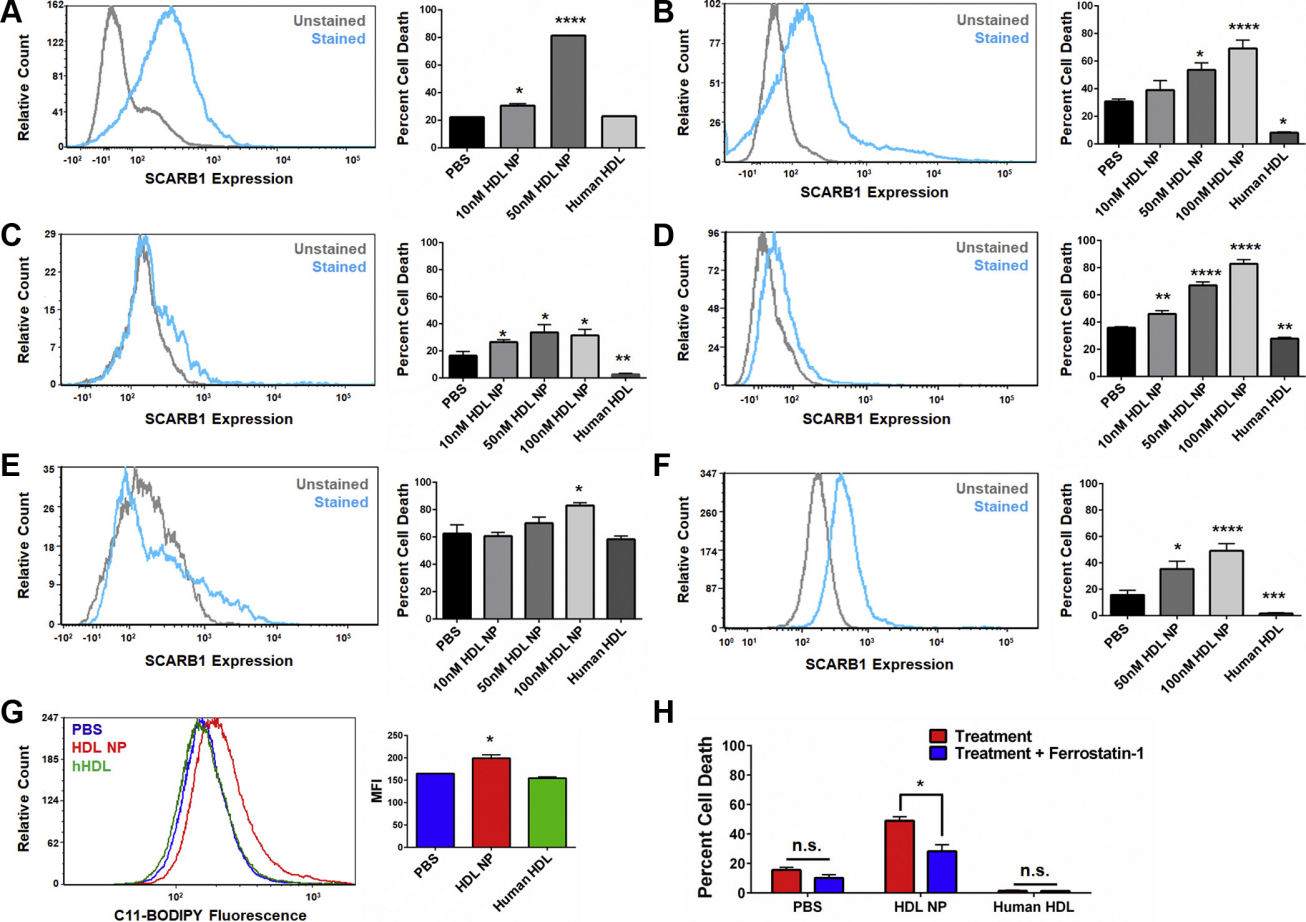
**HDL NPs induce cell death in primary B cell lymphoma cells obtained from patients.***A–F*. SCARB1 expression (flow cytometry, left) and viability (Annexin V/PI staining, right) of primary B cell lymphoma cells isolated from patients with follicular lymphoma and large B cell lymphoma and treated with HDL NPs or human HDL for 72 h (*A–E*) or 120 h (*F*). *A–D*. Follicular lymphoma. *E*. T cell–rich large B cell lymphoma. *F*. DLBCL isolated from ascites fluid. ∗*p* < 0.05 vs. PBS. ∗∗*p* < 0.01 vs. PBS. ∗∗∗*p* < 0.001 vs. PBS. ∗∗∗∗*p* < 0.0001 vs. PBS. *G*. Cell death assay (Annexin V/PI staining) for DLBCL cells from ascites fluid treated with PBS, HDL NPs (100 nM), or human HDL (100 nM), with or without ferrostatin-1 (1 μM) for 120 h. ∗*p* < 0.05 vs treatment. *H*. Flow cytometry for C11-BODIPY fluorescence in DLBCL from ascites fluid following treatment with PBS, HDL NPs (100 nM), or human HDL (100 nM) for 72 h. ∗*p* < 0.05 vs PBS control. DLBCL, diffuse large B cell lymphoma; HDL NPs, high-density lipoprotein–like nanoparticles; PBS, phosphate buffered saline.

To best determine potential clinical relevance, we investigated expression of SCARB1 and whether HDL NPs induce cell death in primary B cell lymphoma cells derived from fresh patient tissue. Primary cells were isolated from patients with a suspected diagnosis of lymphoma after obtaining informed consent under a Northwestern University (NU) Institutional Review Board–approved protocol (STU00208941; CSRC-1343). Ultimately, patients were found to have the following diagnoses: FL (Fig. 6, *A–D*), large cell lymphoma (T cell–rich B cell lymphoma; Fig. 6, *E*), DLBCL (isolated from ascites fluid; Fig. 6, *F*), and non-GC DLBCL (Fig. S9, *B*). Cells were selected for CD19 expression to enrich the biopsy tissue samples for lymphoma cells. This resulted in a mixed population of normal B cells and B cell lymphoma cells. As we have previously shown, normal B cells do not express SCARB1 (26). Accordingly, flow cytometric analysis of the patient samples demonstrated varying levels of SCARB1 expression, as mentioned, likely the result of the mix of normal and malignant B cells (Fig. 6, *A–F*; Fig. S9, *B*).

All of the primary patient samples were incubated with HDL NPs and assayed for cell death. Cell death as a result of HDL NP exposure varied among the patient samples depending upon the lymphoma subtype and relative level of SCARB1 expression. HDL NPs dose-dependently induced cell death in the four FL, T cell–rich B cell lymphoma, and ascites-isolated DLBCL patient samples (Fig. 6, *A–F*; Fig. S9, *C*). Addition of human HDLs had either no effect or significantly rescued the cells from cell death in culture (Fig. 6, *A–F*; Fig. S9, *C*). In the non-GC (ABC) DLBCL sample, HDL NPs did not induce cell death at the concentrations tested (10 and 50 nM; Fig. S9, *B*) even with strong SCARB1 expression. This finding is consistent with our prior published work using HDL NPs as monotherapy for ABC DLBCL (23). Collectively, these data confirm that HDL NP efficacy against B cell lymphoma cell lines can be replicated in primary human SCARB1-positive B cell lymphoma cells *ex vivo*.

To quantify if HDL NPs induce ferroptosis in the primary B cell lymphoma cells, DLBCL cells isolated from ascites fluid were assayed for lipid peroxide accumulation and viability following treatment with HDL NPs and/or ferrostatin-1. HDL NP treatment led to an increase in measured lipid peroxide accumulation, while human HDL treatment did not (Fig. 6, *G*). Correspondingly, addition of ferrostatin-1 reduced HDL NP–induced cell death (Fig. 6, *H*). Taken together, these results demonstrate that the ability of HDL NPs to induce ferroptosis in malignant cells is not restricted to immortalized cell lines. HDL NPs induce ferroptosis in primary lymphoma cells obtained from patients with lymphoma.

## Discussion

The cholesterol-poor HDL NP targets SCARB1 in cholesterol uptake–dependent lymphoma cells. HDL NP binding to SCARB1 results in a switch from a baseline dependence on cholesterol uptake and high GPX4 expression to one favoring de novo cholesterol biosynthesis, which is accompanied by reduced expression of GPX4. As GPX4 is absolutely required by the cancer cell to reduce the burden of membrane lipid peroxides (42), this metabolic switch leaves the cancer cell particularly vulnerable to the accumulation of oxidized membrane lipids and cell death through a mechanism consistent with ferroptosis.

Our data demonstrate that gene and protein expression of GPX4 is reduced after HDL NP binding to the high-affinity receptor for cholesterol-rich high-density lipoproteins, SCARB1. Gene array and RT-qPCR data support that HDL NPs mediate reduced levels of GPX4 by reducing transcription. Six different transcription factors have been shown to play a role in regulating GPX4 expression [reviewed in (43)], including negative regulation by SREBP-1a [sterol response element binding protein-1a (44)] and positive regulation by NF-κB (45). We have previously shown that B cell lymphoma cell cholesterol depletion increases the activation of SREBP-1a, which increases de novo cholesterol biosynthesis (23). Additionally, in a different model system and with HDL NPs slightly chemically modified from those used in this study, HDL NP treatment dose-dependently reduced LPS-induced activation of nuclear factor kappa-light-chain-enhancer of activated B cells (46). More studies are required in order to make definitive conclusions regarding which transcription factor(s) are critical to HDL NP efficacy.

Our Western blot data show a profound reduction in GPX4, which suggest posttranslational mechanisms that might further reduce GPX4. Certainly, in this regard, there is precedent in the literature that products of lipid metabolism, in general, and intermediate metabolites of cholesterol biosynthesis, in particular, are involved in regulation of GPX4 stability (16). Interestingly, inhibition of de novo cholesterol biosynthesis using statins did not reduce GPX4 expression or induce ferroptosis (47), suggesting that inhibition of de novo cholesterol biosynthesis is protective against ferroptosis and critically highlights the difference in mechanism between statins and HDL NPs. Further studies are required to better understand mechanisms through which the HDL NP therapy regulates GPX4 stability.

Pathways involving intermediates in the cholesterol biosynthesis pathway are interesting in the context of the ALK+ ALCL (SR-786, SUDHL1) and U937 cell lines because of their shared inability to synthesize cholesterol owing to enzymatic blockade induced by hypermethylation or mutation, respectively. Our data show that HDL NP treatment increased expression of de novo cholesterol synthesis genes and reduced expression of *GPX4.* In theory, this could serve to even more drastically increase intermediates in the cholesterol biosynthesis pathway that may serve an antioxidant function, but would only be effective at preventing ferroptosis in the presence GPX4. While there have been significant contributions related to intermediates in the cholesterol biosynthesis pathway and their impact on GPX4 and ferroptosis (8, 10, 11, 47), more work is needed here. The HDL NPs provide a unique tool for further mechanistic studies.

We have also observed that HDL NP binding to SCARB1 results in alterations in cell membrane lipid raft microdomains (36), often sites of concentrated cell membrane second-messenger signaling cascades, such as the case with BCR (37). We have demonstrated that HDL NPs synergize with inhibitors of specific receptor tyrosine kinases to induce cell death in ABC DLBCL (23). Thus, it is also possible that HDL NPs targeting SCARB1 modulate important downstream second-messenger signaling pathways in cholesterol uptake–dependent lymphomas and that this also contributes to ferroptosis.

Our data suggest that investigation of HDL NPs in cholesterol auxotrophic cell lines is warranted, despite the fact that these cells can uptake cholesterol *via* LDLs binding the LDLR. SCARB1 is expressed by the three auxotrophic cell lines investigated, suggesting that both the LDLR and SCARB1 play a role in supplying the cells with cholesterol. Indeed, as has been demonstrated for native HDLs (39, 48), our synthetic HDL NPs can bind to both SCARB1 and the LDLR. However, it was only through SCARB1 binding that cell death was induced in both the B and T cell lymphoma cell lines. As mentioned above, and by contrast to LDL/LDLR, HDL binding to SCARB1 has been linked to intracellular signaling pathways, including the prosurvival PI3K/AKT pathway (49). A recent report suggests that a decrease in GPX4 expression correlated with decreased phosphorylation of AKT (50). As such, it is possible that engagement of HDL NPs to SCARB1 not only prevents cholesterol influx but also disrupts membrane-anchored prosurvival signaling pathways that may, ultimately, impact GPX4 expression. Regardless, targeted inhibition of cholesterol uptake by synthetic nanoparticles built upon an inert core appears to be an important target in certain cholesterol auxotrophic or cholesterol uptake–dependent cancers.

In this study, and in many previous studies using the HDL NP as systemic therapy in animal models, we have noted a lack of toxic side effects (23, 26, 27, 38, 51, 52). We also reported that HDL NP treatment of primary human hepatocytes and macrophages, the two most abundant normal cell types that express SCARB1, did not result in toxicity and that each of the normal cell types tightly regulated cholesterol homeostasis when treated with the HDL NP or native human HDL (26). Coupled with data demonstrating the potent toxicity of HDL NPs toward lymphoma cancer cells that have been reprogrammed to depend upon cholesterol uptake and GPX4 expression to prevent ferroptosis, our working hypothesis to explain the lack of toxicity is that normal cells do not have the same oxidative burden as the cancer cells and the normal cells are able to maintain plasticity with regard to cholesterol metabolism. New data support this conclusion as HDL NP treatment in normal human hepatocytes did not result in a reduction in GPX4 expression or the accumulation of lipid peroxides.

In conclusion, we report that HDL NPs target SCARB1 in cholesterol uptake–dependent and GPX4-dependent lymphoma cells revealing an apparent reciprocal oncometabolic response favoring an increase in de novo cholesterol biosynthesis at the expense of GPX4 expression. Ultimately, this results in the death of the cancer cells by ferroptosis while sparing normal, healthy cells. As this metabolic profile is not unique to lymphomas, it is possible that HDL NPs may have translational relevance in a range of cholesterol-addicted cancers that are sensitive to cell death by ferroptosis following GPX4 depletion.

## Experimental procedures

### Cell lines

The Ramos (RRID: CVCL_0597), SUDHL4 (CVCL_0539), Raji (CVCL_0511), Daudi (CVCL_0008), SUDHL6 (CVCL_2206), Namalwa (CVCL_0067), Jurkat (CVCL_0367), SUDHL1 (CVCL_0538), SR-786 (CVCL_1711), and U937 (CVCL_0007) human cell lines were obtained from ATCC and were used within 3 months of receipt and/or resuscitation. ATCC uses short tandem repeat (STR) profiling to authenticate their cell lines prior to shipping. For SUDHL4 cells, Charles River Laboratories was contracted to test for *mycoplasma* contamination prior to use in animal experiments. All cell lines were cultured in RPMI (Corning) 1640 supplemented with 10% fetal bovine serum and 1% penicillin/streptomycin at 37 °C in a humidified, 5% CO_2_ incubator.

### HDL NP synthesis

The HDL NPs were synthesized and quantified as previously described (53). Five-nanometer-diameter citrate-stabilized gold nanoparticles (AuNPs) were surface-functionalized with apolipoprotein A-I, followed by addition of the phospholipids, 1,2-dipalmitoyl-*sn*-glycero-3-phosphoethanolamine-N-[3-(2-pyridyldithio)propionate] (PDP PE) and 1,2-dipalmitoyl-*sn-*glycero-3-phosphocholine (DPPC). The HDL NPs were purified using the KrosFlo TFF (Tangential Flow Filtration) system with a 50-kDa cutoff polyethersulfone module. The concentration of HDL NPs was calculated using UV-Vis spectroscopy and Beer’s law.

To synthesize fluorescently labeled HDL NPs, the intercalating dye DiI was added at a 1 μM final concentration during the phospholipid addition step. Purification and quantification of the fluorescently labeled HDL NPs were conducted as described above.

### HDL NP binding assay

Ramos, SUDHL4, SR-786, SUDHL1, U937, and Jurkat cells were incubated with DiI HDL NPs (10 nM) in standard culture media for 2 h at 37 °C in the presence or absence of a SCARB1 blocking Ab (Novus Biologicals; 1:100; RRID: AB_1291690), LDLR blocking Ab (Novus Biologicals; 1:100 dilution; AB_2135126), and/or the rabbit IgG isotype control Ab (Novus Biologicals; 1:100). Cells were washed once with 1 ml of ice-cold fluorescent activated cell sorter (FACS) buffer (PBS, 1% bovine serum albumin, 0.1% sodium azide) and resuspended in 500 μl of ice-cold FACS buffer prior to flow cytometric analysis (BD LSR II Fortessa). Data were analyzed using the FCS Express software (De Novo Software).

### Microarray analysis

Ramos cells were treated with HDL NPs (40 nM), human HDL (40 nM), or PBS for 48 h prior to RNA isolation using the RNeasy Mini kit (Qiagen). RNA samples were converted to cDNA libraries by the NU Genomics Core facility and were then run on the Illumina HT-12 microarray. A total of three biological replicates were run for each condition. Data were analyzed by the Genomics Core facility, with a fold change of >1.5 or <−1.5, and a *p* value <0.05 considered significant. Microarray data are available at the National Center for Biotechnology Information Gene Expression Omnibus under accession number GSE98028.

### Western blot analysis

Western blots were conducted as previously described (23). Blots were imaged using the Azure 3000 imager. The SCARB1 Ab (Abcam, RRID: AB_882458; 1:1000), the GPX4 Ab (Abcam, AB_941790; 1:5000), the HMGCS1 Ab (Cell Signaling Technologies, AB_2799216; 1:1000), the β-actin Ab (Cell Signaling Technologies, AB_2223172; 1:3000), and a secondary Ab (goat anti-rabbit HRP, Bio-Rad, AB_11125142; 1:2000) were used in these experiments.

### RT-qPCR analysis

Ramos, SUDHL4, SUDHL1, SR-786, and U937 cells were treated with HDL NPs (20 nM, 50 nM), human HDL (hHDL; 50 nM), or PBS for up to 72 h, and RNA isolated using the RNeasy Mini kit (Qiagen). In all cases, hHDLs were added at an equimolar concentration to HDL NPs based upon protein concentration, as previously described (26). RNA samples (500 ng RNA/30 μl reaction) were reverse transcribed using a TaqMan Reverse Transcription kit, and qPCR was performed using TaqMan Gene Expression Assays (Life Technologies) on a BioRad CFX-Connect iCycler. Samples were standardized to β-actin, and relative expression was calculated using the ΔΔCt method. Biological triplicates were run for each condition.

### C11-BODIPY assay for lipid peroxidation

Ramos, SUDHL4, SUDHL1, SR-786, and U937 cells (2.5 × 10^5^ cells/ml) were treated with HDL NPs (50 nM) or PBS for 24, 48, or 72 h. Following treatment, C11-BODIPY (1 μM final concentration; Thermo Fisher Scientific) was added to each well and the cells were incubated for 30 min at 37 °C, 5% CO_2_. The cells were then washed twice with 1 X PBS, resuspended in ice-cold FACS buffer and C11-BODIPY fluorescence in the FITC channel quantified using the BD LSR II Fortessa flow cytometer. Data were analyzed using the FCS Express software.

### Cell death (MTS) assay

MTS assays (CellTiter; Promega) were conducted as described previously (23, 26). The Ramos, SUDHL4, Raji, Daudi, Namalwa, SUDHL6, and Jurkat cells were plated at a density of 2 × 10^5^ cells/ml and cultured for 72 h prior to assay. SUDHL1, SR-786, and U937 cells were plated at a density of 5 × 10^4^ cells/ml and cultured for 5 days prior to MTS assay. The SCARB1 blocking, LDLR blocking, and isotype control antibodies were added at dilutions of 1:1000 to 1:250. Ferrostatin-1 and deferoxamine (DFO) were obtained from Sigma Aldrich and added at a final concentration of 1 μM. MTS values were standardized to PBS control.

### Lentiviral vector transfection of GPX4 expression plasmid

The GPX4 expression plasmid SC324086 was obtained from Origene. The plasmid was expanded in One Shot BL21 (DE3) chemically competent *Escherichia coli* cells. Ampicillin (100 μg/ml) was used for selection. The plasmid was purified using the Qiagen QIAfilter Plasmid Maxi Kit. The resultant plasmid prep was packaged into a lentiviral vector by NU’s Gene Editing Transduction & Nanotechnology Core in the Skin Biology & Diseases Resource-Based Center.

Ramos cells were plated at a density of 2.5 × 10^5^ cells/ml and infected with the GPX4 lentivirus or empty construct by centrifugation at 3000× rpm for 90 min at room temperature (25 °C), as described previously (54). The cells were then cultured for 48 h before antibiotic selection was initiated. G418 was added at a concentration of 100 μg/ml. Following 3 to 7 days of selection, Ramos cells were then assayed for GPX4 expression by Western blot analysis and RT-qPCR, and cell viability following HDL NP treatment by MTS assay, as described above.

### Primary human hepatocyte culture

Primary human hepatocytes were obtained from Lonza and cultured in Hepatocyte Culture Media (HCM), consisting of Hepatocyte Basal Media (Lonza) supplemented with Lonza’s HCM SingleQuot, as described previously (26). Briefly, cells were plated at a density of 1 × 10^5^ cells/ml and allowed to attach overnight prior to treatment with HDL NPs. Cells were treated with 20 nM or 50 nM HDL NPs, 50 nM human HDL or PBS for 72 or 120 h. Lysates were prepared using the mammalian protein extraction reagent (MPER) cell lysis buffer, and Western blotting analyses for GPX4 and β-actin conducted as described above. For the C11-BODIPY assay, the primary human hepatocytes were treated for 72 h prior to addition of the C11-BODIPY reagent and subsequent analysis by flow cytometry.

### Tumor xenograft model

All animal work was conducted in accordance with NU’s Institutional Animal Care and Use Committee and Center for Comparative Medicine facilities under an approved animal protocol (NU Institutional Animal Care and Use Committee IS00002415). Severe combined immunodeficiency–beige mice (4- to 6-week-old; Charles River) were used for the SUDHL4 tumor xenograft study. Flank tumors were initiated using 1 × 10^7^ SUDHL4 cells per mouse. Tumors were allowed to reach ∼100 mm^3^ before HDL NP treatments began. Based on their initial tumor volumes, mice were randomly divided into two groups, PBS (100 μl) and HDL NPs (100 μl of 1 μM NPs). Treatments (intravenous) were administered three times per week for 1 week. Tumors were then harvested, and single-cell suspensions generated by mechanically dissociating the tumors and passing the cells through a 70-micron filter. C11-BODIPY (1 μM final concentration) was added to a fraction of the resultant cell suspension (1 × 10^6^ cells), and flow analysis was carried out as described above. RNA was isolated from the remainder of the cells to quantify GPX4 expression by RT-qPCR, as described above.

### Human tissue analysis

Archived, formalin-fixed, paraffin-embedded tissue sections were analyzed from patients with large B cell lymphoma and follicular lymphoma. All samples were deidentified of all information other than final diagnosis. A total of 104 follicular lymphoma and 49 DLBCL archival samples were obtained and stained for SCARB1 expression. Immunohistochemical staining of the sections was performed using a monoclonal SCARB1 Ab (Abcam, AB_882458; 1:100 dilution) by the Pathology Core at the Robert H. Lurie Comprehensive Cancer Center of NU. Liver and thymus specimens were utilized as positive and negative controls, respectively. Bright-field images were captured at 10× and 40× magnifications.

### Primary lymphoma cell isolation and analysis

Primary lymphoma cells were isolated from excisional biopsies from patients with suspected B cell lymphomas, in accordance with an NU Institutional Review Board–approved protocol (STU00208941; CSRC-1343) and abiding by the principles set forth in the Declaration of Helsinki. Samples were deidentified of all information other than eventual final diagnosis, provided to investigators approximately 1 week after excisional biopsy. A total of seven samples were analyzed, with diagnoses of follicular lymphoma (N = 4), T cell–rich DLBCL (N = 1), DLBCL isolated from ascites fluid (N = 1), and non-GC (activated B cell [ABC]) DLBCL (N = 1). The excised tissue was washed with 1 × PBS and mechanically dissociated using two 18-G needles. The solution was then passed through a 70-micron filter, washed with 1 × PBS, and centrifuged at 400× *g* for 5 min at room temperature. The cells were resuspended in RPMI 1640 with L-glutamine and 25 mM Hepes (4-(20hydroxyethyl)-1-piperasineethanesulfonic acid) containing 10% fetal bovine serum and 1% PenStrep. Cells were cultured at 1 × 10^6^ cells/ml for 1 to 2 days. Following culture, CellSep Human CD19^+^ selection kit (Stem Cell Technologies) was used to enrich for CD19^+^ cells. Flow cytometry was used to quantify SCARB1 and CD19 expression. Data were analyzed using the FCS Express software.

To quantify cell death, CD19^+^-enriched primary lymphoma cells were cultured in the presence of PBS, human HDL, or HDL NPs for 72 h. The cells were then collected, stained with Annexin V-FITC and propidium iodide (PI) (Invitrogen), and run on the BD LSR II flow cytometer (BD Biosciences). Data were analyzed with the FCS Express software. The total dead cells were analyzed as the total Annexin V–positive cell population, in both the PI-positive and PI-negative gates (Annexin V–FITC^+^/PI^-^) and (Annexin V–FITC^+^/PI^+^) cells. To quantify ferroptosis in primary DLBCL cells, CD19+-enriched cells were cultured in the presence of PBS, human HDL (100 nM), or HDL NPs (100 nM) in the presence or absence of ferrostatin-1 (final concentration of 1 μM). Viability was quantified as described above. Lipid peroxide accumulation was measured using C11-BODIPY, as described above.

### Statistical analyses

Each *in vitro* cell line experiment was repeated three times. Based on our previous experience with the SUDHL4 xenograft model, three mice were used per group. For the primary lymphoma sample assays, each sample was run once, with four or more per treatment group. One-way ANOVAs and student’s *t* test were used to determine statistical significance, where appropriate. All statistical analyses were calculated using the GraphPad Prism software.

## Data availability

The microarray data presented here are available via the National Center for Biotechnology Information Gene Expression Omnibus website under accession number GSE98028. All other data presented are contained within the manuscript.

## Conflict of interest

The authors declare the following conflict of interest: C. S. T., K. M. M., and L. I. G. are cofounders of a biotechnology company that licensed the HDL NP technology from Northwestern University. The remaining authors, J. S. R., A. L., A. E. C., S. Y., T. T., J. M., A. C., R. K., and A. B., declare no competing interests.
