# Supplementary material for: Targeted reduction of cholesterol uptake in cholesterol-addicted lymphoma cells blocks turnover of oxidized lipids to cause ferroptosis
Source: J Biol Chem. 2020 Nov 24;296:100100. doi: 10.1074/jbc.RA120.014888 (PMC7949030; doi:10.1074/jbc.RA120.014888)
Supplement: Supplementary file 1 — Figs. S1–S9 [file mmc1.pdf]

Supporting Materials to Accompany:

Targeted reduction of cholesterol uptake in cholesterol-addicted lymphoma cells blocks turnover of oxidized lipids to cause ferroptosis

Jonathan S. Rink[1,2,3], Adam Lin[1,3], Kaylin M. McMahon[2,4], Andrea E. Calvert[2,4], Shuo Yang[1,3], Tim Taxter[3,5], Jonathan Moreira[1], Amy Chadburn[6], Amir Behdad[3,5], Reem Karmali[1,3], C. Shad Thaxton[2,3,4,7,*] and Leo I. Gordon[1, 3,*]

1: Division of Hematology/Oncology, Department of Medicine, Northwestern University Feinberg School of Medicine, Chicago, IL, USA 60611
2: Simpson Querrey Institute for BioNanotechnology, Northwestern University, Chicago, IL, USA 60611
3: Robert H Lurie Comprehensive Cancer Center of Northwestern University, Chicago, IL, USA 60611
4: Department of Urology, Northwestern University Feinberg School of Medicine, Chicago, IL USA 60611
5: Department of Pathology, Northwestern University Feinberg School of Medicine, Chicago, IL, USA 60611
6: Department of Pathology, Weill Cornell Medical Center, New York, NY, USA 10021
7: International Institute for Nanotechnology (IIN), Northwestern University, Evanston, IL, USA, 60208

***Corresponding authors:**

C. Shad Thaxton, MD PhD
Department of Urology
Feinberg School of Medicine
Northwestern University
303 E. Chicago Ave., Tarry 16-703
Chicago, IL 60611
cthaxton003@northwestern.edu

Leo I. Gordon, MD
Division of Hematology/Oncology
Feinberg School of Medicine
Northwestern University
676 N St. Clair, Suite 850
Chicago, IL 60611
l-gordon@northwestern.edu

Running Title: *Targeting Scavenger Receptor Type B1 to Induce Ferroptosis*

Keywords: Cholesterol; High-Density Lipoprotein (HDL); Nanotechnology; Lipid Peroxidation; Lymphoma; Scavenger Receptor Type B1 (SCARB1); Glutathione Peroxidase 4 (GPX4); Ferroptosis

Supplementary Figure S1

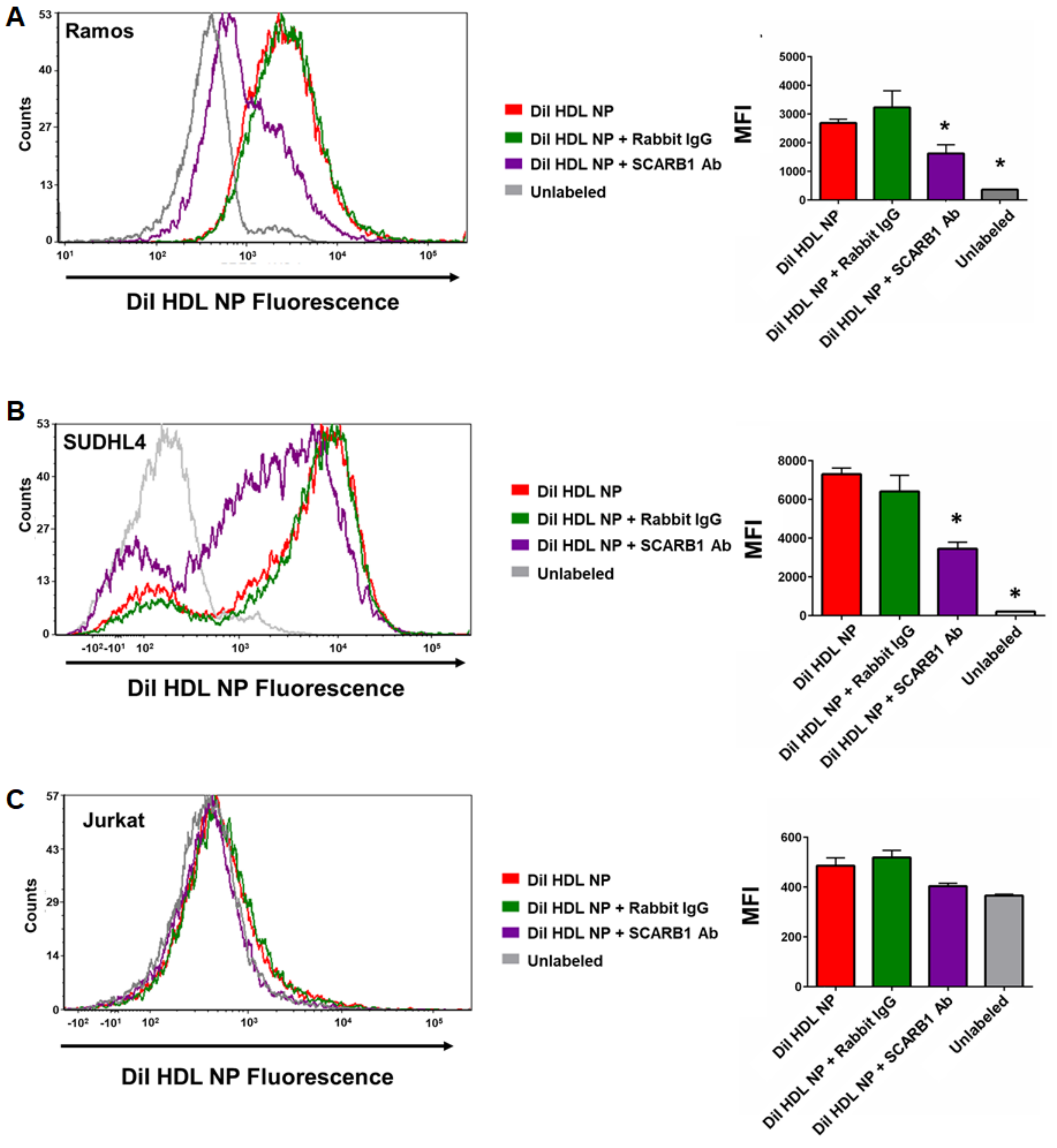

Supplementary Figure S1: HDL NPs Bind to SCARB1 on B Cell Lymphoma Cells. Flow cytometric analysis of the binding of DiI HDL NPs to Ramos (A), SUDHL4 (B) and Jurkat cells (C) treated with the SCARB1 blocking antibody (1:100), Rabbit IgG isotype control antibody (1:100), or untreated. **Left-**Representative histogram of DiI HDL NP treated cells. **Right-**Median fluorescent intensity. *p<0.05 vs. DiI HDL NPs.

Supplementary Figure S2

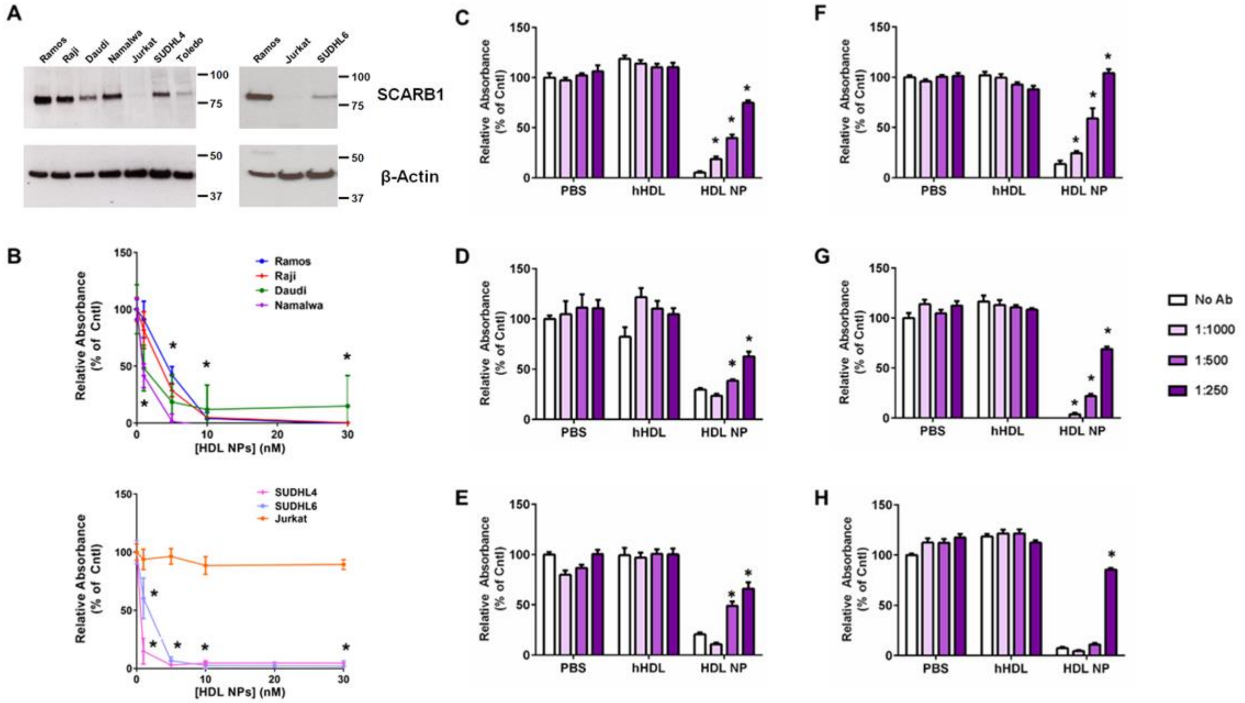

Supplementary Figure S2: HDL NPs Target SCARB1 and Induce Cell Death in GC DLBCL and BL Cell Lines. A. Western blot analysis for SCARB1 in BL (Ramos, Raji, Daudi, Namalwa) and GC DLBCL (SUDHL4, SUDHL6) cell lines. The SCARB1-negative T cell leukemia/ lymphoma cell line Jurkat was used as a negative control. β-actin was used as a loading control. B. MTS assays for BL (Top) and GC DLBCL (Bottom) cell lines treated with increasing concentrations of HDL NPs. N=6 for all conditions. *p<0.05 vs. PBS control. C-H. Cell survival as assessed by MTS assays of SCARB1 blocking antibody treated Ramos (C), Raji (D), Daudi (E), Namalwa (F), SUDHL4 (G), and SUDHL6 (H) cells exposed to human HDL (hHDL) or HDL NPs (10nM). N=6 for all conditions. *p<0.05 v. No Ab.

Supplementary Figure S3

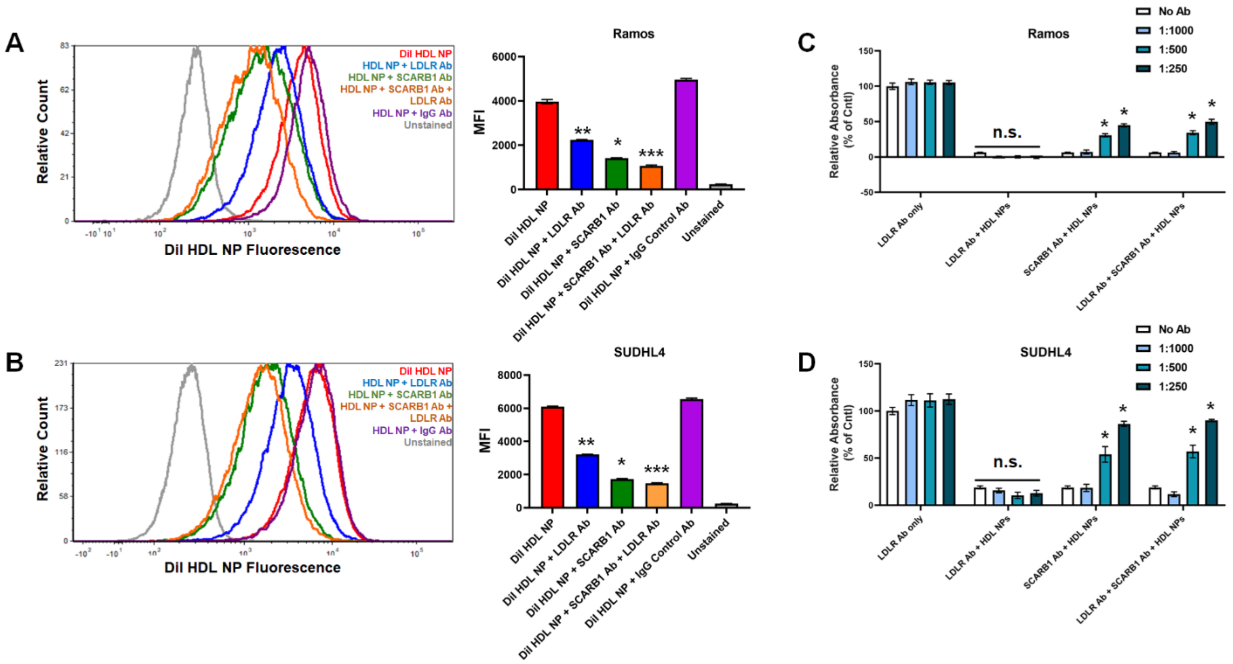

Supplementary Figure S3: SCARB1 and LDL Receptor (LDLR) Blocking and Cell Death Studies in Ramos and SUDHL4. A, B. Flow cytometric analysis of the binding of DiI HDL NPs to Ramos (A) and SUDHL4 (B) cells treated with the SCARB1 blocking antibody (1:100), the LDLR blocking antibody (1:100), a combination of SCARB1 and LDLR blocking antibodies, rabbit IgG isotype control antibody (1:100), or untreated. **Left-**Representative histogram of DiI HDL NP treated cells. **Right-**Median fluorescent intensity. *p<0.05 vs. DiI HDL NP, DiI HDL NP + LDLR Ab, and DiI HDL NP + SCARB1 Ab + LDLR Ab. **p<0.05 vs. DiI HDL NP, DiI HDL NP + SCARB1 Ab, and DiI HDL NP + SCARB1 Ab + LDLR Ab. ***p<0.05 vs. DiI HDL NP, DiI HDL NP + SCARB1 Ab, and DiI HDL NP + LDLR Ab. C, D. MTS assays of Ramos (C) and SUDHL4 (D) cells treated with HDL NPs (10nM) and the LDLR blocking antibody, SCARB1 blocking antibody or a combination of the two blocking antibodies. *p<0.05 vs. No Ab. No statistically significant differences were observed between the HDL NPs + SCARB1 Ab and HDL NPs + SCARB1 Ab + LDLR Ab groups at each of the four antibody dilutions.

Supplementary Figure S4

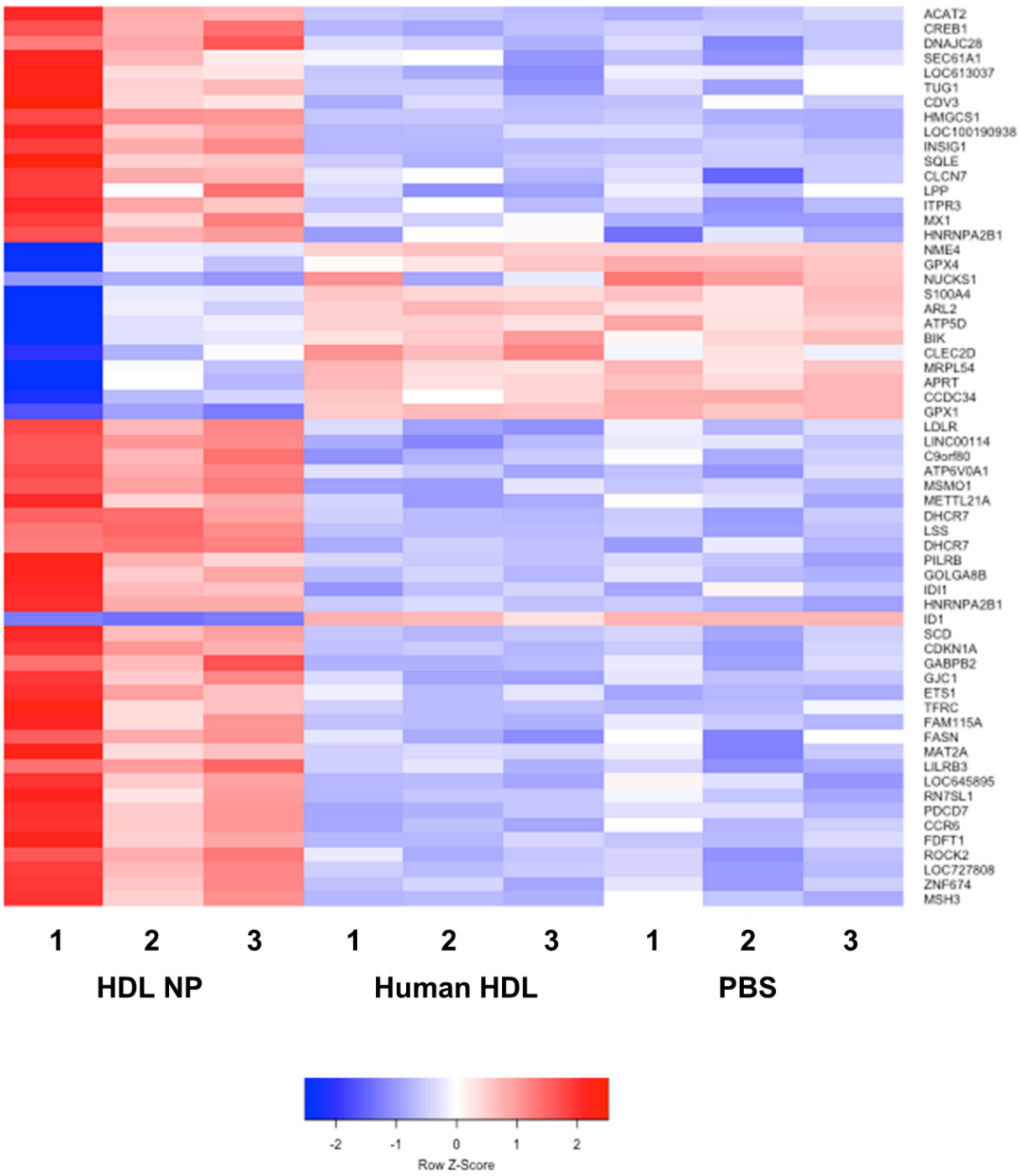

Supplementary Figure S4: HDL NPs Alter Gene Expression in Ramos Cells. Ramos cells were treated with PBS, human HDL (40nM) or HDL NPs (40nM) for 48 hours prior to RNA extraction for analysis by Illumina's HT-12 microarray. All genes listed had a fold change of >1.5 or <-1.5 vs. PBS control, with a corresponding p value less than 0.05. Each treatment was run in biological triplicates, indicated by the number 1, 2 and 3 on the heatmap.

Supplementary Figure S5

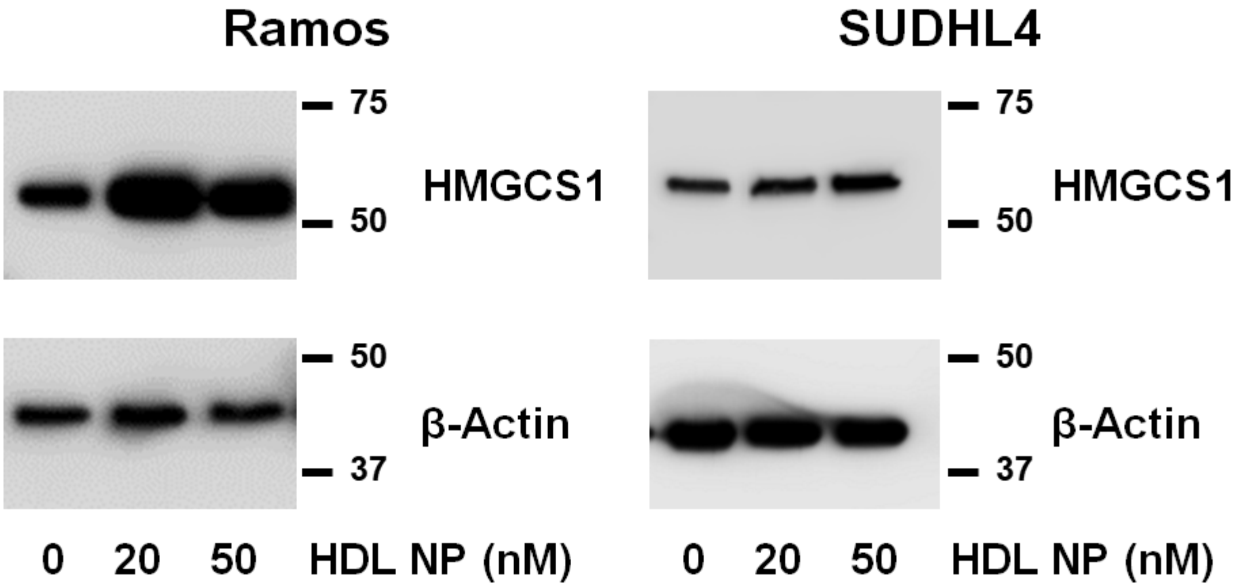

Supplementary Figure S5: HDL NPs Increase HMGCS1 Expression in Ramos and SUDHL4 Cells. Ramos and SUDHL4 cells were treated with PBS or HDL NPs (20nM or 50nM) for 48 hours prior to protein lysate collection and western blot analysis.

Supplementary Figure S6

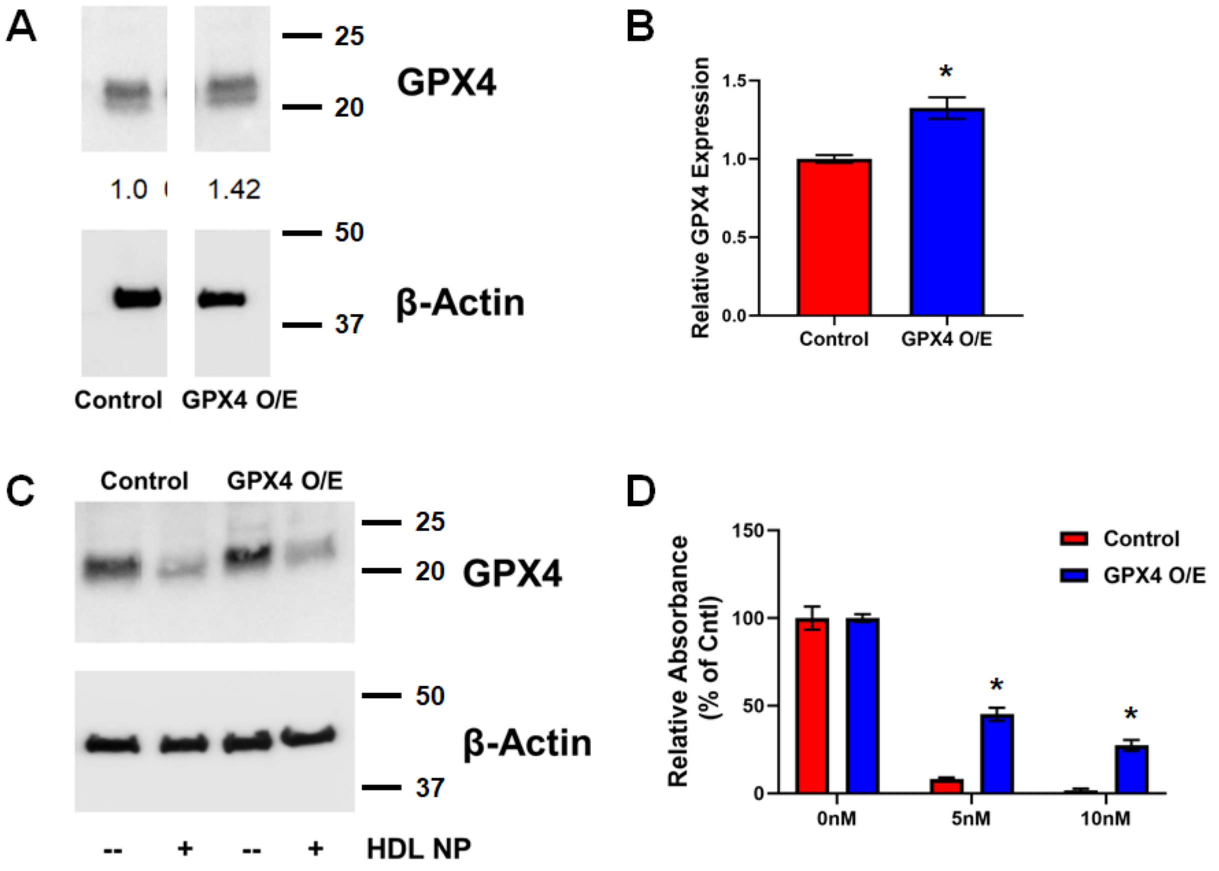

Supplementary Figure S6: Over-Expression of GPX4 protects Ramos cells from HDL NP-Induced Cell Death. A. Western blot analysis of Ramos cells infected with control or GPX4 over-expression (GPX4 O/E) plasmids. B. RT-qPCR verification of GPX4 overexpression compared with control. *p=0.011 vs. Control. C. Western blot for GPX4 in Control and GPX4 O/E cells treated with PBS or 5nM HDL NPs for 48 hours. β-Actin was used as the loading control. D. MTS assay for Control and GPX4 O/E cells treated with 0nM, 5nM or 10nM HDL NPs for 72 hours. *$p<0.05$ vs. Control cells at the same HDL NP concentration.

Supplementary Figure S7

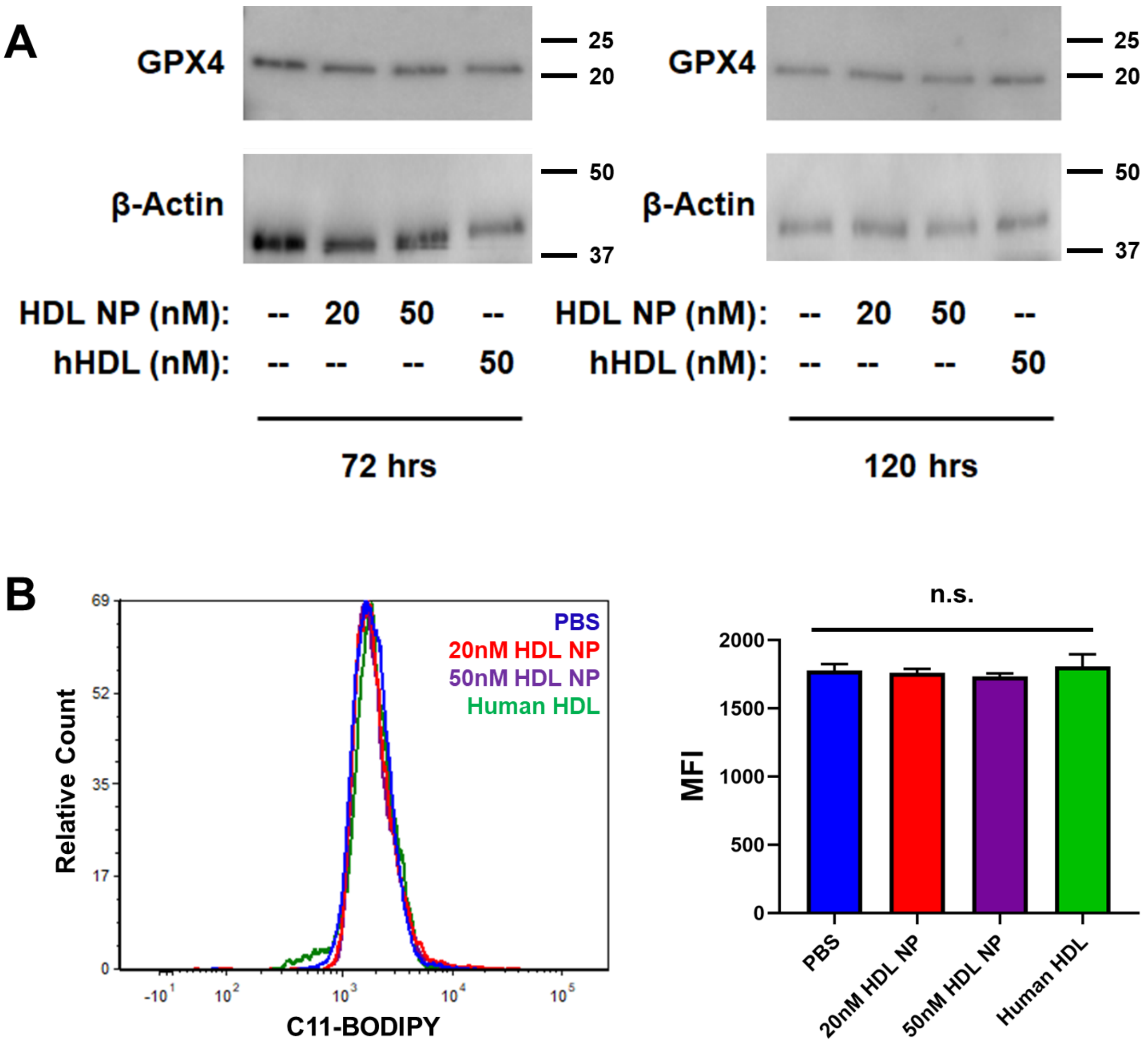

Supplementary Figure S7: GPX4 Expression and Lipid Peroxide Levels in Primary Human Hepatocytes after HDL NP treatment. A. Western blot for GPX4 from primary human hepatocytes treated with HDL NPs (20nM, 50nM), human HDL (50nM) or PBS for 72 or 120 hours. β-actin was used as the loading control. B. C11-BODIPY flow cytometric analysis of primary human hepatocytes treated with HDL NPs (20nM, 50nM), human HDL (50 nM) or PBS for 72 hours.

Supplementary Figure S8

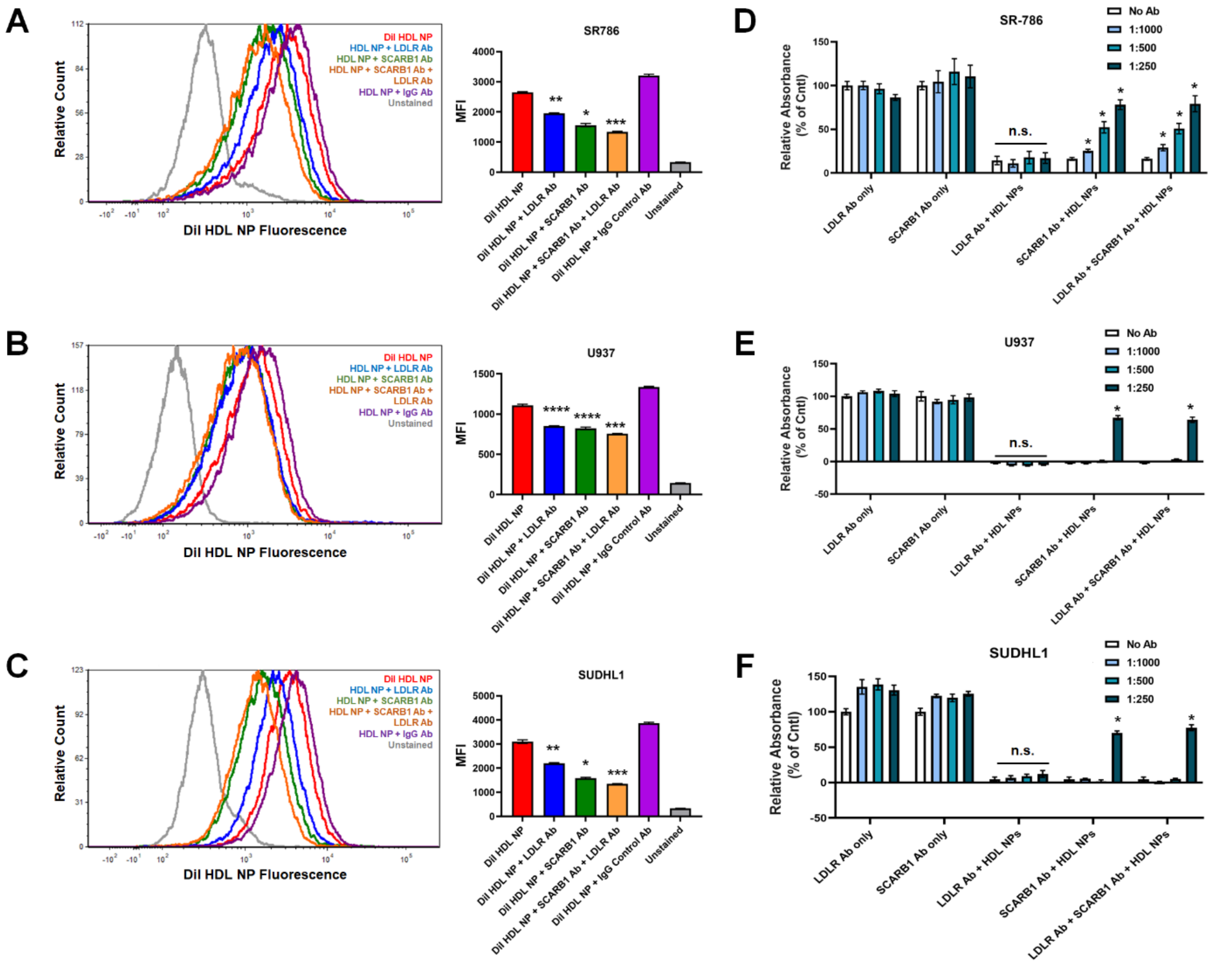

Supplementary Figure S8: SCARB1 and LDL Receptor (LDLR) Blocking and Cell Death Studies in Cholesterol Auxotrophic Cell Lines. A-C. Flow cytometric analysis of the binding of DiI HDL NPs to SR-786 (A), U937 (B) and SUDHL1 cells (C) treated with the SCARB1 blocking antibody (1:100), the LDLR blocking antibody (1:100), a combination of SCARB1 and LDLR blocking antibodies, rabbit IgG isotype control antibody (1:100), or untreated. **Left-** Representative histogram of DiI HDL NP treated cells. **Right-**Median fluorescent intensity. *p<0.05 vs. DiI HDL NP, DiI HDL NP + LDLR Ab, and DiI HDL NP + SCARB1 Ab + LDLR Ab. **p<0.05 vs. DiI HDL NP, DiI HDL NP + SCARB1 Ab, and DiI HDL NP + SCARB1 Ab + LDLR Ab. ***p<0.05 vs. DiI HDL NP, DiI HDL NP + SCARB1 Ab, and DiI HDL NP + LDLR Ab. ****p<0.05 vs. DiI HDL NPs, and DiI HDL NPs + SCARB1 Ab + LDLR Ab. D-F. MTS assays of SR-786 (D), U937 (E) and SUDHL1 (F) cells treated with HDL NPs (25nM) and the LDLR blocking antibody, SCARB1 blocking antibody or a combination of the two blocking antibodies. *p<0.05 vs. No Ab. No statistically significant differences were observed between the HDL NPs + SCARB1 Ab and HDL NPs + SCARB1 Ab + LDLR Ab groups at each of the four antibody dilutions.

Supplementary Figure S9

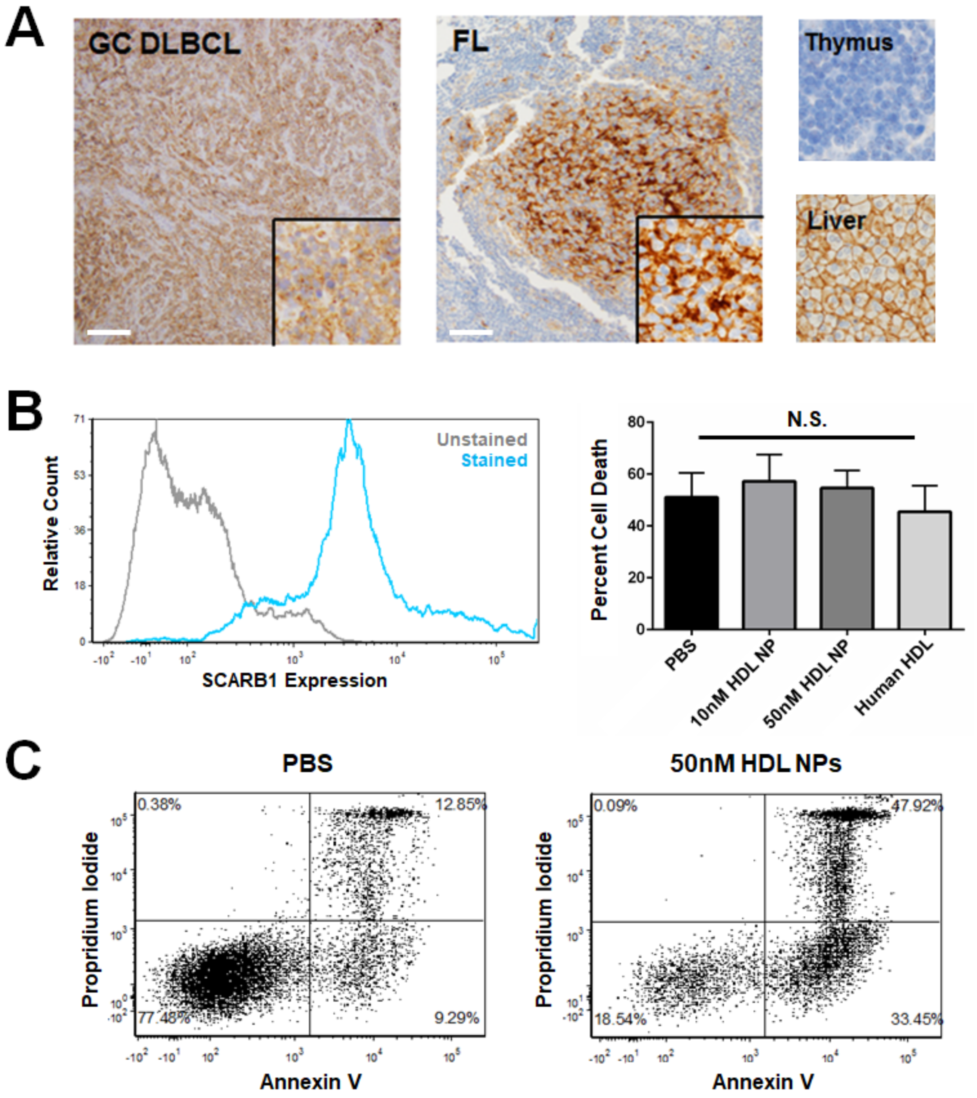

Supplementary Figure S9: SCARB1 Expression and HDL NP Efficacy Against Primary Lymphoma Cells. A. Formalin-fixed, paraffin embedded tissue samples, obtained at the time of diagnosis were sectioned and stained for SCARB1 (brown staining). Representative positive staining of DLBCL (N = 49; 26.5% of which displayed ≥10% of malignant cells positive for SCARB1) and FL (N = 104; 10.6% of which displayed ≥10% of malignant cells positive for SCARB1). Thymus and Liver sections (right) are presented as negative and positive staining controls, respectively. Images were taken at 10X magnification. Scale bar = 200µm. Insets and control images were taken at 40X magnification. B. Flow cytometric analyses of SCARB1 and viability of HDL NP treated primary DLBCL cells isolated from a patient with non-GC (ABC) DLBCL. C. Representative dot plots of Annexin V/ PI stained primary FL cells treated with PBS or 50nM HDL NPs.
